# Pediatric acute myeloid leukemia tumor composition predicts patient outcomes at diagnosis and reveals mechanisms of resistance to chemotherapy

**DOI:** 10.21203/rs.3.rs-4669225/v1

**Published:** 2026-01-23

**Authors:** Mohammad Javad NajafPanah, Alexandra M Stevens, Michael J Krueger, Max Rochette, Sohani Sandhu, Lana Kim, Sridevi Addanki, Josh Cooper, Hua-Sheng Chiu, Jessica Epps, Sonal Somvanshi, Barry Zorman, Maria Rodriguez Martinez, Marianna Rapsomaniki, Susanne Unger, Burkhard Becher, Joanna S. Yi, Tsz-Kwong Man, Michele L Redell, Pavel Sumazin

**Affiliations:** 1 Texas Children’s Cancer Center, Baylor College of Medicine, Houston, TX, USA; 2 Biomedical Informatics & Data Science, Yale School of Medicine, New Haven, CT, USA; 3 CHUV-FBM Biomedical Data Science Center, University of Lausanne, Lausanne, Switzerland; 4 Institute of Experimental Immunology, University of Zurich, Zurich, Switzerland

## Abstract

Although most pediatric acute myeloid leukemia (pAML) patients achieve complete remission with standard-of-care chemotherapy, overall outcomes are poor, and 40% will eventually relapse. Improved methods for risk assessment at diagnosis and alternative therapies are needed to improve outcomes for these patients. Toward these objectives, we characterized the clonal composition of pAMLs, identifying subclones that expand or transform between diagnosis and relapse. We further showed that the abundance of these expanding and transforming subclones in diagnostic samples is predictive of patient outcomes and, similarly, predicts response to chemotherapy and targeted therapies in patient samples and patient-derived xenograft models. Moreover, gene expression programs previously associated with pAML chemoresistance are recurrently elevated in these predictive subclones. Consequently, we propose a novel strategy for improving pAML risk prediction at both diagnosis and during therapy that combines the detection of outcome-predictive tumor subclones in pAML blood or bone marrow with cytogenetic biomarkers and residual disease assessment. Critically, we showed that this combination dramatically improved risk prediction, including for patients who achieve complete remission after chemotherapy. Moreover, through our analyses of outcome-predictive pAML subclones, we identified potential personalized targeted therapies for pAML patients based on the composition of their tumors.

## INTRODUCTION

Despite the availability of aggressive therapies, including high-dose chemotherapy regimens and stem cell transplantation, over 40% of pediatric acute myeloid leukemia (pAML) patients relapse, including 30% of those who are considered low-risk at diagnosis^1–3^. At relapse, pAMLs are chemoresistant and patient outcomes are poor; however, the early identification of chemoresistant cells can help clinicians tailor therapies to improve outcomes for high-risk patients^4^. Currently, risk-prediction methods are based on the identification of recurrent, prognostically predictive genetic variants at diagnosis^1^ and the detection of measurable residual disease (MRD) after one round of chemotherapy^5^. However, for most patients, the ability to predict relapse based on the genetic composition of cancer cells at diagnosis is limited. Moreover, although some stem cell gene profiles indicative of chemoresistant pAMLs have been characterized, these signatures help predict outcomes for only a small fraction of the patient population^3^.

Many leukemias, including pAMLs, are highly heterogeneous, with individual patients often harboring multiple types of pAML cells (subclones) that exhibit a variety of DNA alterations, gene expression signatures, protein markers, and treatment responses. Critically, some pAML subclones are successfully eradicated by standard chemotherapy treatments, whereas others survive at undetectable levels during treatment. High-resolution RNA and protein profiling technologies can be leveraged to characterize rare pediatric and adult AML subclones^3,6^. Single-cell RNA sequencing (scRNA-seq) profiles of adult AML patients revealed malignant cells with monocyte-like signatures that expressed T cell suppressing genes^7^, and scRNA-seq analysis of chronic myeloid leukemia samples identified a response-predictive signature^8^. Another single-cell profiling study of five paired AML samples collected at diagnosis and relapse identified common pathways associated with disease progression, including enhanced fatty acid oxidation and amino acid metabolism^9^. However, while these studies informed on the biology of pAML, they neither improved current pAML risk prediction nor proposed new therapeutic strategies for high-risk pAML patients^3^.

Here, we analyzed scRNA-seq expression profiles of paired diagnosis–relapse pAML samples obtained from 13 pAML patients together with local and publicly available transcriptomic profiles from unpaired diagnosis or relapse pAML samples and healthy donor bone marrow aspirates. Our analysis revealed transcriptionally distinct cell subpopulations, including those predicted to proliferate or transform between diagnosis and relapse. We reasoned that resistance to chemotherapy conferred a selective advantage to these pAML subclones, and consistent with this interpretation, we found that their abundance in diagnostic samples was significantly predictive of patient outcomes and could be leveraged in conjunction with current technologies to improve outcome prediction at both diagnosis and remission. Moreover, in patient-derived xenograft models (PDXs), these outcome-predictive pAML subclones were predictive of model response to cytarabine chemotherapy.

Analysis of the expression profiles of outcome-predictive subclones further identified gene expression signatures associated with pAML chemoresistance and relapse, including genes that were co-expressed with FMS-related receptor tyrosine kinase 3 (*FLT3*) and cyclin-dependent kinase 6 (*CDK6*), which are associated with early hematopoietic differentiation states, and the MYC and oxidative phosphorylation pathways. However, as noted in prior studies, gene sets alone were predictive of outcomes for only a small subset of patients^3,10^. Instead, our results suggested that the detection of high-risk cells at diagnosis is most predictive of both patient outcomes and chemotherapy responses and that the combination of tumor-composition analyses with current standard cytogenetic biomarkers and residual disease assessment significantly improved risk prediction at diagnosis and during treatment. Finally, our results identified potential subclone-specific therapies and indicated that most high-risk pAML subclones mimic FLT3 and CDK6 activation. Consequently, we argued that therapies—including retinoic acid agonists and FLT3 inhibitors—should be chosen based on tumor composition.

## RESULTS

### Merging single-cell RNA expression profiles of bone marrow samples across pAML patients

We first sought to identify pAML subclones that are common across multiple patients and that can be used to distinguish cancer from non-cancer immune cells and to identify chemoresistant cancers. Toward this goal, we characterized cancer and other immune cells that recurrently populate the bone marrow and blood of pAML patients by integrating longitudinal scRNA-seq profiles of samples taken at diagnosis and relapse with profiles from unpaired pAML and healthy bone marrow samples. In total, we assessed the profiles of 13 paired samples, an unpaired relapse sample, and healthy bone marrow samples from two donors. Patients with paired samples included six patients from Texas Children’s Hospital (TCH1–6) and seven from the Children’s Oncology Group (COG) biorepository that were collected as part of the Therapeutically Applicable Research to Generate Effective Treatments (TARGET) cohort (PAPZCL, PARBIU, PAUMTZ, PAUNSV, PAIVAT, PAVBFN, and PAVTLN). The additional unpaired relapse sample was from a COG patient (PAWNPU). All patients were treated according to the guidelines of the COG Phase 3 clinical trial, AAML1031^11^; clinical and molecular characteristics of our pAML cases are described in Table S1. We further included publicly available profiles from eight diagnostic pAML samples and four healthy donors provided by Bailur *et al.*^12^. Because some samples, including those from Bailur *et al*., were flow-sorted for CD45 positivity to enrich for blasts and myeloid cells, we profiled a sample from one healthy donor with and without sorting to evaluate and control for the effects of cell sorting on gene expression profiles.

**Figure 1.**
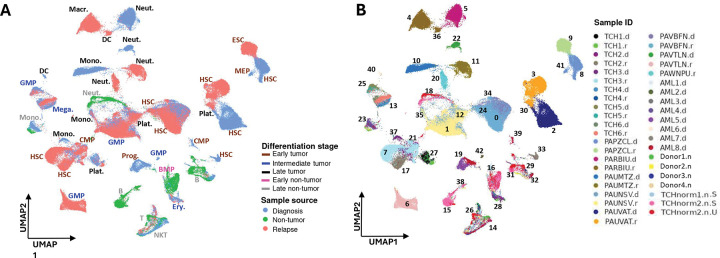

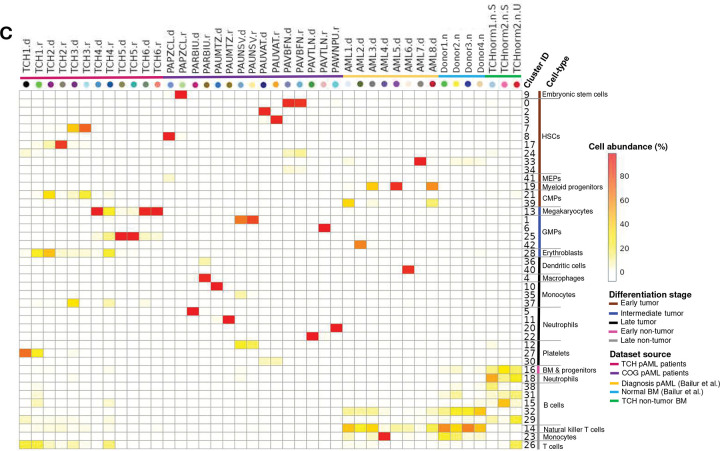
Merged single-cell RNA sequencing (scRNA-seq) profiles in pediatric acute myeloid leukemia (pAML patients). **(A)** Uniform manifold approximation and projection (UMAP) visualization of scRNA-seq expression profiles from samples collected at pAML diagnosis (blue) and relapse (red), as well as from healthy bone marrow donors (non-tumor, green). Clusters were annotated based on SingleR-inferred cell types, and their associated differentiation stage was visualized using a five-color font palette. **(B)** RNA expression profiles of cells from 42 samples—including 13 diagnosis–relapse pAML sample pairs, one relapse and eight diagnosis unpaired samples, and seven bone marrow samples from healthy donors—grouped into 43 distinct clusters. Diagnosis, relapse, and non-tumor samples are marked with .d, .r, and .n extensions, respectively. **(C)** A comprehensive schematic summary of the cell clusters identified in the above analyses, indicating their samples of origin, inter-sample abundance, inferred cell types and differentiation stages, and data source (e.g., TCH, Texas Children’s Hospital; COG, Children’s Oncology Group; Bailur et al., 2020); see also Table S2. Macr: Macrophages; Neut: Neutrophils; DC: Dendritic cells; GMP: Granulocyte-monocyte progenitor cells; CMP: Common myeloid progenitor cells; Mega: Megakaryocytes; Mono: Monocytes; HSC: Hematopoietic stem cells; MEP: Megakaryocyte erythroid progenitor cells; ESC: Embryonic stem cells; Plat: Platelets; Prog: Myeloid progenitors; Ery: Erythroid cells; NKT: Natural killer T cells; T: T cells; B: B cells.

Attempts to integrate all collected scRNA-seq profiles using either Seurat tools^13^ or Harmony^14^ suggested that pAMLs are highly heterogeneous. Whereas non-cancer cells had similar transcriptional profiles across patients, pAML cells clustered mainly by donor. Both sample merging, wherein the profiles of each cell and cluster are normalized independently and not altered to match, and integration, in which expression profiles are altered to correct for deviations across assays or batches, produced similar clustering trends. Namely, non-cancer cells, including T cells, natural killer T (NKT) cells, B cells, monocytes, and erythroid cells, had similar transcriptomes across patients and clustered together (Figures 1 and S1, and Table S2). In contrast, most cancer cell clusters were composed of cells exclusively from one sample or patient. Based on these observations, we chose to compare pAML cell profiles one patient at a time, using three normal samples from two healthy donors to help distinguish between cancer and non-cancer cells (Figure S2 and Table S3). We then compared analysis results across patients—a meta-analysis approach avoids the direct comparisons of molecular profiles across patients. Cell cluster uniform manifold approximation and projection (UMAP) visualizations of each paired diagnosis–relapse profile are shown in Figure S2. Note that, the cell types of each subclone reported in Figure 1 were inferred using singleR^15^ based on a combination of expression profiles from multiple cell-type databases; see Methods. Using this approach, we categorized cells derived from pAML samples as embryonic stem cells, hematopoietic stem cells (HSCs), common myeloid progenitors (CMPs), megakaryocytic–erythroid progenitors, myeloid progenitors, megakaryocytes, granulocyte–monocyte progenitors, erythroblasts, dendritic cells, macrophages, monocytes, neutrophils, and platelets.

### More than half of the diagnostic samples contained pAML subclones that expanded at relapse

**Figure 2.**
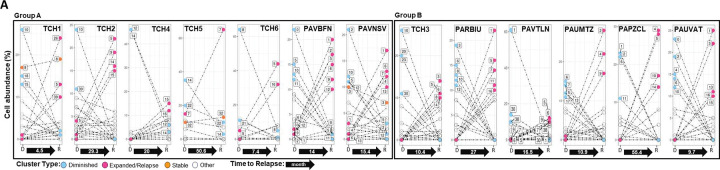

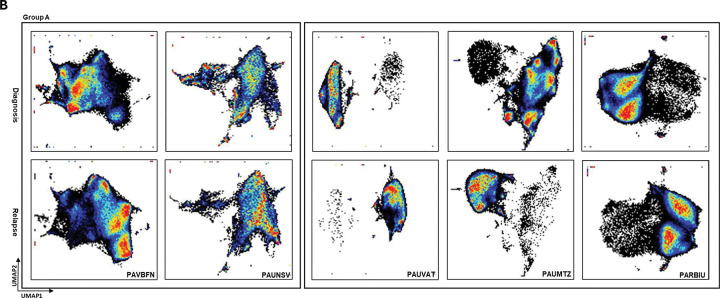

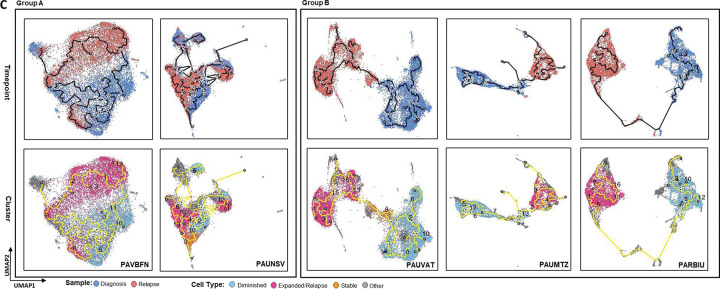
Longitudinal single-cell molecular profiles identify patients with expanding and transforming pAML subclones. **(A)** Patients were classified based on the presence (Group A) or absence (Group B) of expanding pAML subclones in diagnostic samples. Line plots map the estimated relative abundance of subclones in the RNA profiles of samples collected at diagnosis and relapse from two Group A and three Group 3 patients; expanded cells are present in both groups, and time to relapse (months) is indicated. **(B)** UMAP visualization of cytometry by time of flight (CyTOF) profiles and **(C)** trajectory plots based on scRNA-seq profiles of representative pAML pairs from Group A and B patients. UMAP color gradients represent cell density for protein expression profiles in (B) and sample/cell type for RNA expression profiles in (C); predicted trajectories are illustrated with solid lines.

We next identified pAML subclones with cells in both diagnosis and relapse samples for patients with paired samples, hypothesizing that these cell populations are more likely to be resistant to therapy (Figure S3). Cells in the diagnosis samples of seven of our thirteen paired pAMLs transcriptomically matched cells in their paired relapse samples. The remaining six pAML diagnosis–relapse pairs did not contain cells with longitudinally matching transcriptomic signatures (Figures 2A and S2). To distinguish between them, we classified pAML subclones in diagnostic samples into three categories: *expanded*, *stable*, and *diminished* to indicate those identified at higher, similar, or lower relative abundance at relapse, respectively. pAML subclones in relapse samples that did not transcriptomically match cells in their diagnostic counterparts were identified as *relapse* cells (or subclones). We used these terms to refer to cells, clusters, or subclones, where subclones may be composed on multiple computationally inferred clusters with similar expression profiles.

We posit that expanded pAML subclones have gained a competitive advantage during or after treatment and are more likely to be chemoresistant, whereas diminished subclones were either sensitive to chemotherapy or transformed during treatment to acquire chemoresistance (Figure S3). To further investigate this model, we classified individuals with and without identified expanded cells as *Group A* and *Group B* patients, respectively; note that time to relapse (Figures 2A and S4) was not significantly different between the groups. This grouping—based on the analysis of scRNA-seq profiles—was confirmed by cytometry by time of flight (CyTOF) profiling of two Group A and three Group B diagnosis-relapse pairs (Figure 2B). Interestingly, trajectory analysis identified potential diminished-to-relapse cell transformation trajectories for cell populations in both Group A and Group B patients (Figures 2C and S4). In total, analysis of our 13 paired diagnosis–relapse samples identified 49 expanded cell clusters, three stable cell clusters, and 45 diminished cell clusters, 14 of which were predicted to be transforming. Cell population frequencies in paired samples are given in Tables S3 and S4.

### Identification of recurrently upregulated genes in expanded and relapse subclones

**Figure 3.**
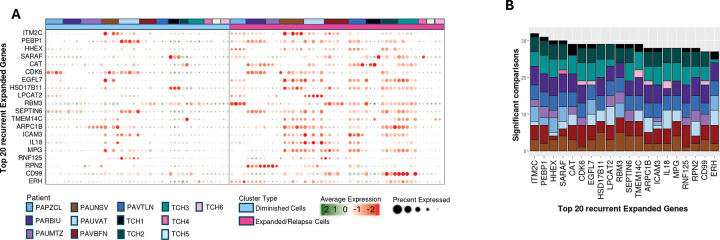

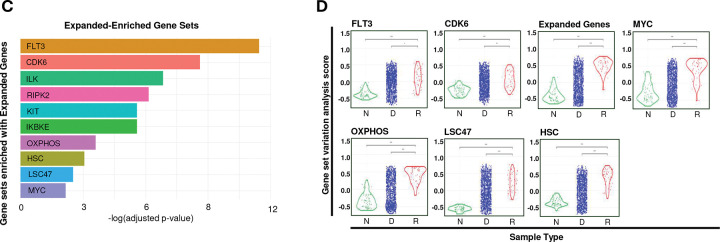

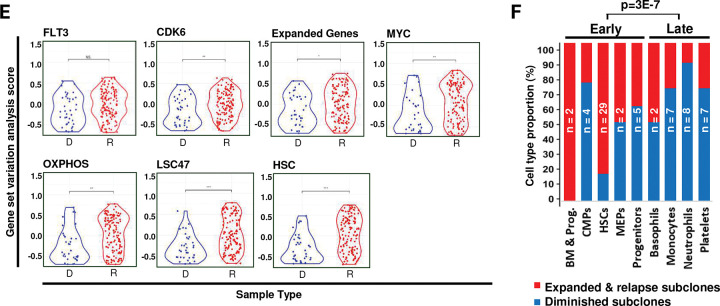
Recurrently upregulated genes and pathways in expanded and relapse pAML subclones. **(A)** Average expression of the top 20 recurrently upregulated genes (Expanded Genes) in expanded (cell clusters showing greater abundance at relapse vs. diagnosis) and relapse (subclones emerging at relapse) cells. Genes were ranked based on the number of expanded and relapse vs. diminished cell-cluster comparisons in which they show significant upregulation. **(B)** The number of comparisons in which Expanded Genes were significantly upregulated across patient profiles. **(C)** Gene sets and pathways that are significantly enriched with Expanded Genes (i.e., Expanded–Enriched Gene Sets), including genes that are co-expressed with Fms-related receptor tyrosine kinase 3 (FLT3), cyclin-dependent kinase 6 (CDK6), integrin-linked kinase (ILK), receptor-interacting serine/threonine kinase 1 (RIPK), KIT proto-oncogene (KIT), and inhibitor of nuclear factor kappa-B subunit epison (IKBKE), as well as pathways including Hallmark oxidative phosphorylation (OXPHOS), hematopoietic stem cell (HSC), leukemia stem cell populations gene set (LSC47), and MYC targets. **(D, E)** Gene Set Variation Analysis (GSVA) revealed upregulation of Expanded–Enriched Gene Sets pAML, particularly in relapse samples, from the (D) Therapeutically Applicable Research to Generate Effective Treatments (TARGET) AML and (E) St. Jude’s AML datasets; N, bone marrow aspirates from non-cancer donors; D, diagnosis samples; R, relapse samples. **(F)** The proportion of expanded and diminished subclones as a function of pAML differentiation.

Differential expression analysis comparing signatures in expanded, stable, and relapse subclones to those in diminished subclones in each patient identified 140 genes recurrently upregulated in expanded and relapse subclones (referred to here as Expanded Genes); see Methods and Table S5. Expression profiles of the 20 Expanded Genes with the strongest evidence for recurrent upregulation (i.e., significant upregulation in the largest number of expanded/relapsed vs. diminished cell comparisons) are visualized in Figure 3A (Table S6). Figure 3B shows the cumulative counts of subclone comparisons in which each of these 20 Expanded Genes were significantly upregulated. Gene Set Enrichment Analysis (GSEA) with all 140 Expanded Genes identified multiple enriched gene sets, including *FLT3*– and *CDK6*–co-expressed genes (Figure 3C). Other gene sets and pathways enriched among Expanded Genes included Hallmark reactive oxygen species pathway and oxidative phosphorylation genes, HSC genes^3^, and a set of 47 genes that were previously identified as enriched in leukemia stem cell populations (LSC47)^16^. We refer to these as Expanded–Enriched Gene Sets and note that these gene sets have been previously implicated in AML relapse^16–20^. Gene Set Variation Analysis (GSVA) of Expanded–Enriched Gene Sets in 1,435 TARGET and 170 St. Jude pAML RNA-seq profiles confirmed that these gene sets are significantly upregulated in pAMLs, particularly in samples from relapsed patients (Figure 3D, E).

As previously observed^3^, GSEA and GSVA analyses using the Expanded–Enriched Gene Sets LSC47 and HSC were predictive of overall survival (OS) for FLT3-internal tandem duplication (ITD)–positive patients; and Expanded, LSC47, and HSC were also predictive for FLT3-ITD–negative patients with mixed-lineage leukemia (MLL) rearrangements. However, gene sets were not predictive for other cytogenetic categories or the FLT3-ITD–negative patient population (Figures S5–S10); note that FLT3-ITD–positive AAML1031 patients were evaluated separately because their treatment differed from that of FLT3-ITD–negative patients. Interestingly, analysis of pAML cell differentiation confirmed that expanded/relapse subclones, which are hypothesized to be more chemoresistant, were enriched in less-differentiated pAML subclones, including CMPs, HSCs, and progenitor cells (Figure 3F).

### Risk-predictive pAML subclone abundance in diagnostic samples

**Figure 4.**
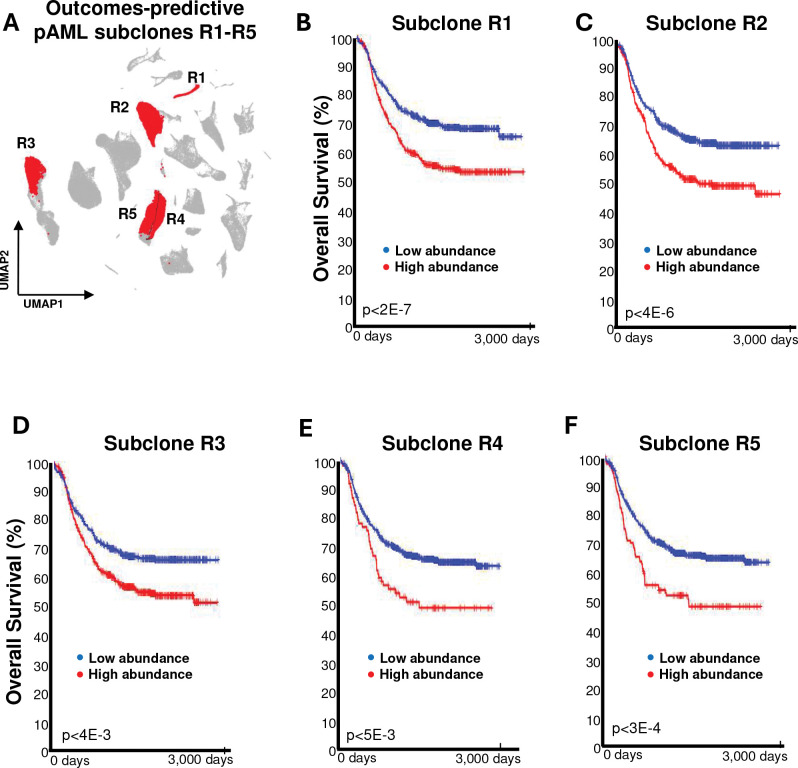

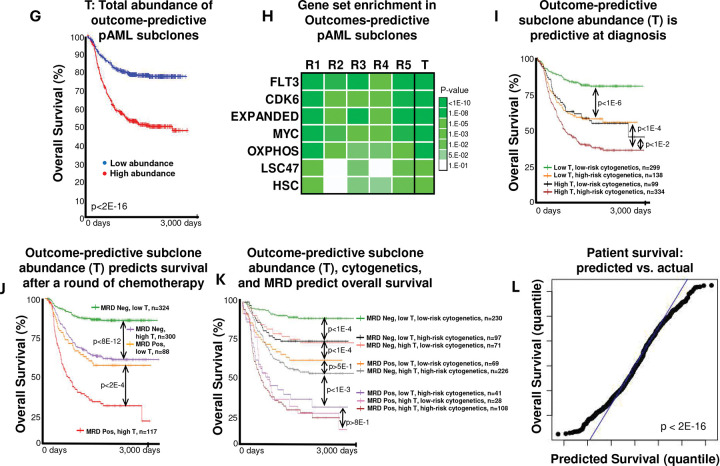
Expanded and transforming pAML subclones predict patient outcomes. **(A)** Highly correlated pAML cell clusters were merged using the SQUID framework, reducing the 138 patient-specific clusters to 90 pAML subclones. Expanding and transforming subclones were tested for prognostic ability using SQUID-based deconvolution of 870 TARGET-profiled FLT3-ITD–negative diagnostic pAML samples from patients enrolled on AAML1031, identifying five subclones (R1–R5 in the displayed UMAP) that were significantly predictive of prognosis after multiple-testing correction. **(B–G)** Kaplan–Meier survival analyses for patients with higher and lower median inferred subclone abundance; panels B–F show results for individual subclones R1–R5, and (G) reflects their total abundance (T). **(H)** Enrichment of Expanded–Enriched Gene Sets and pathways in cell populations comprising each subclone, R1–R5, and their aggregate, T. (**I**) Kaplan–Meier survival analysis of pAML patients classified as low- or high-risk based on cytogenetic profiles who have a low or high T at diagnosis. (**J**) Kaplan–Meier survival analysis of pAML patients positive or negative for measurable residual disease (MRD) who have a low or high T. (**K**) Kaplan–Meier survival analysis indicated that combining T with cytogenetics-based risk assessment and MRD dramatically improved risk prediction. (**L**) Q–Q plot comparing the overall and predicted survival suggests significantly improved accuracy for a linear regression function based on the combination of outcome-predictive cells, MRD after one round of chemotherapy, and cytogenetics relative to predictive functions based on any single feature or pair of features (see also Figure S14).

We next evaluated whether the abundance of expanding and transforming pAML subclones in the diagnostic samples of TARGET-profiled FLT3-ITD–negative pAML patients is risk predictive; see Methods. Subclone abundance was estimated from the bulk RNA-seq profiles of TARGET diagnostic samples using the SQUID framework^6^. In total, five subclones (R1–R5 in Figures 4 and S11) were predictive of both OS and event-free survival (EFS) at an adjusted p<0.01. Subclones R1, R2, and R4 were composed of expanded clusters, whereas R3 and R5 contained diminished clusters with evidence for transformation after diagnosis—clusters 13 and 2 in the profiles of PAUMTZ.d and PAUVAT.d, respectively. Moreover, the total inferred abundance of R1–R5 subclones (referred to as T in Figure 4 and hereafter) in TARGET cases was significantly more predictive of OS and EFS than that of any individual subclone (p<0.01 by F-test). As expected, Expanded–Enriched Gene Sets were upregulated in R1–R5 subclones and in their aggregate (T), including FLT3– and CDK6–co-expressed genes (Figure 4H); note that T refers to the total (sum) abundance of R1–R5 cells.

We further compared the risk-prediction capabilities of our outcome-predictive pAML subclones with those of other methods in clinical use. Current risk-prediction methods combine cytogenetic profiles at diagnosis and MRD detection during treatment^21,22^. Analysis of diagnostic samples from TARGET-profiled FLT3-ITD–negative pAML patients confirmed that tumors with core binding factor fusions (*CBFB::MYH11* and *RUNX1::RUNX1T1*) and those with normal karyotypes were associated with better outcomes than tumors with *KMT2A* rearrangements or other cytogenetics (Figure S12A). Therefore, we classified the former as low-risk and the latter as high-risk for our outcome-prediction analyses. Application of the more extensive cytomolecular classification used in the AAML1831 trial to the AAML1031 cohort was also significantly predictive of patient outcomes (Figures S12B); see Methods. Overall, MRD at the end of Induction 1 was the most predictive individual parameter (Figure S12C).

We then evaluated whether combining the inferred total abundance of our outcome-predictive pAML subclones (T) with cytogenetics-based risk prediction or with MRD yielded improved predictive accuracy for OS and EFS. Results show that combining T abundance with cytogenetic classification indeed resulted in improved predictive accuracy, dividing each cytogenetic group into subsets with significantly better and worse outcomes (Figures 4I, S13A, and 13B). Similarly, combining T with MRD-based risk stratification significantly improved the predictive accuracy of MRD for both OS and EFS (Figures 4J, S13C, and S13D). Moreover, when combined with both cytogenetics-based risk prediction and MRD, T abundance significantly improved risk prediction. Of note, T abundance was most valuable for risk assignment when cytogenetics and MRD-based risks conflicted. For instance, high T abundance identified a group of patients (n=28) with low-risk cytogenetics but with positive MRD whose survival was as dismal as that of patients with high-risk cytogenetics and positive MRD.

The inferred total abundance of our outcome-predictive pAML subclones (T) added substantial risk-prediction value for MRD-negative patients with high-risk cytogenetics. Those with low T abundance (n=97) had outcomes similar to patients with low-risk cytogenetics and negative MRD, whereas patients with high T abundance, high-risk cytogenetics, and negative MRD had an intermediate survival rate (Figure 4K, S14A, Table S8). Multivariate Cox proportional hazard model analysis also indicated that after controlling for cytogenetics and MRD, median T abundance remained significantly predictive, exhibiting a hazard ratio of 1.3 (p=3.69e-05; Figure S14B and Table S9).

### Abundance of outcome-predictive subclones predicted PDX model response to chemotherapy

**Figure 5.**
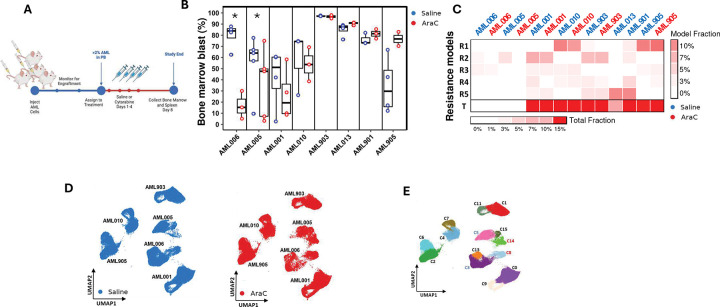

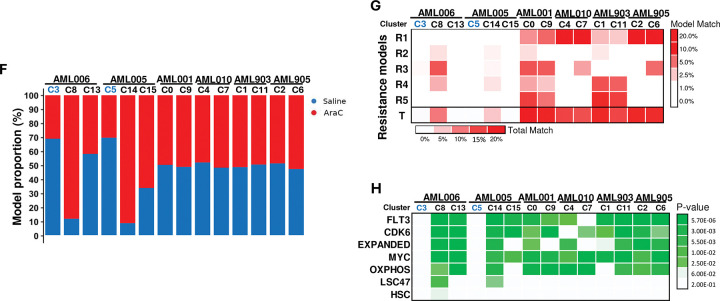

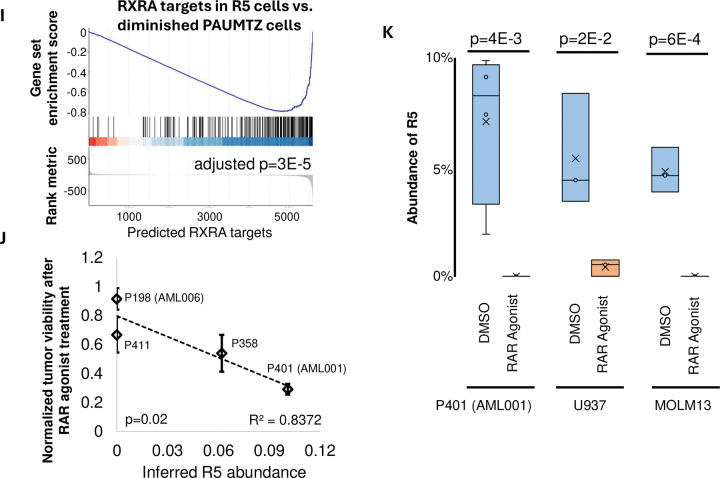
Outcome-predictive subclones also predict response to chemotherapy and a targeted therapy in pAML samples, PDXs, and cell lines. **(A)** PDX populations with proven tumor loads were treated with short (4-day) courses of cytarabine (AraC) chemotherapy or placebo (saline), then euthanized 4 days after the last dose for evaluation of disease burden. **(B)** Bone marrow blast proportions after treatments identified two PDX models (AML006 and AML005) with significant responses to chemotherapy. **(C)** SQUID-based deconvolution of bulk RNA-seq profiles in representative models showed that AML006 and AML005 were the only PDX models with a low—but not zero—abundance of outcome-predictive cells. (**D**) Visualization of the scRNA-seq profiles of AML006 and AML005 and four less chemosensitive models showed cell cluster per model with (**E**) 2–3 subclones per model. (**F**) Analysis of the relative abundance of pAML subclones in AraC- and saline-treated models identified AML006 and AML005 subclones that diminish (C3 and C5) and expand (C8 and C14) after chemotherapy. The abundance of subclones in other models did not vary by treatment. (**G**) Diagonally weighted least squares estimation identified associations between outcome-predictive pAML subclones (R1–R5) and both the expanding subclones of AML006 and AML005 (C8 and C14) and subclones in all other models but not with the diminishing subclones in AML006 and AML005 (C3 and C5). **(H)** GSEA showed upregulation of Expanded–Enriched Gene Sets and pathways in all PDX model subclones, excluding the diminishing subclones of AML006 and AML005. (**I**) Retinoid X Receptor Alpha (RXRA) targets were significantly downregulated in R5 relative to diminishing subclones from the same diagnosis sample. (**J**) R5 abundance was predictive of tumor cell viability in 4 pAML patient samples followed treatment by the retinoic acid receptor agonist tamibarotene when compared to compared to dimethyl sulfoxide (DMSO); s.e.m. shown, patient samples p198 and p401 were used to generate the PDXs AML006 and AML001, respectively. (**K**) Treatments by the retinoic acid receptor agonists tamibarotene (p401, 100 nM, 24h.) or all-trans retinoic acid (ATRA, 1 μM, 72 h., cell lines U937 and MOLM13) significantly reduced the inferred abundance of R5 when compared to DMSO.

To further determine the impact of outcome-predictive pAML subclones, we evaluated tumor responses to cytarabine in eight PDX mouse models from our Pediatric Acute Leukemia Xenograft (PALeX) resource, comparing the proportion of human pAML cells in the bone marrow and spleen of PDX mice after treatment with cytarabine or saline control for 4 days (Figure 5A); see Methods for details. In total, we identified two PDXs with significant responses to cytarabine (AML006 and AML005). Of the remaining six PDXs, two showed some—but not significant—response (AML001 and AML010), and four (including AML903 and AML905) showed no indication of cytarabine sensitivity (Figure 5B); see Table S10 for PDX annotations. Assessment of the cellular composition of cytarabine-treated or saline-treated PDXs, as inferred by SQUID from representative-sample bulk RNA-seq profiling, revealed that the abundance of outcome-predictive pAML subclones (R1–R5 and their total T) was markedly lower in the AML006 and AML005 models (p<5E-6 by *t*-test, Figure 5C). However, all PDXs were predicted to include some representations of these subclones.

We profiled representative PDX models by scRNA-seq to investigate their clonal composition in greater detail. Consistent with our observations in human tumors, scRNA-seq profiles of cytarabine-treated and saline-treated AML006, AML005, AML001, AML010, AML903, and AML905 PDXs revealed PDX-specific molecular signatures (Figure 5D), with 2–3 distinct clusters identified per model (Figure 5E). Interestingly, comparing the composition of each subclone across treatments revealed expanding and diminishing cell populations in PDXs with a significant response to cytarabine (i.e., AML006 and AML005) but not in other PDXs (Figure 5F). Cells from clusters C3 (AML006) and C5 (AML005) showed significantly lower abundance in cytarabine-treated mice compared to controls, while cells from clusters C8 (AML006) and C14 (AML005) showed significantly higher subclone abundance in cytarabine-treated models. Namely, the composition of clusters C8 and C14 expanded by at least 3-fold in cytarabine-treated mice compared to saline controls (p<0.05 by *t*-test). Thus, we concluded that C3 and C5 cells diminished while C8 and C14 expanded after cytarabine treatment, suggesting that these clusters are chemosensitive and chemoresistant pAML subclones, respectively. Interestingly, RNA expression profiles of C8 and C14 cells, like all clusters from the remaining chemoresistant models (excluding the diminished C3 and C5) showed a high degree of similarity to those of our outcome-predictive subclones R1–R5 (Figure 5G); see Methods. This pAML subclone analysis supported our conclusion derived from bulk RNA-seq profiles of AML006 and AML005, indicating that models with significant treatment responses to cytarabine contained both chemosensitive and chemoresistant subclones.

GSEA analysis suggested that all but the diminishing clusters C3 and C5 were enriched for Expanded–Enriched Gene Sets, including FLT3– and CDK6–co-expressed genes (Figure 5H). We note that AML006 and AML005 each included a third cluster, C13 and C15, respectively, with a balanced composition across treatments. These clusters had mixed presentations, with intermediate expression profiles (Figure 5E) and a low abundance of outcome-predictive pAML subclones but upregulated Expanded–Enriched Gene Sets. These clusters also contained fewer cells, with less than 15% of the cells profiled from AML006 and AML005. In comparison, the diminished clusters C3 and C5 each contained over 60% of the cells profiled from AML006 and AML005, whereas the expanded clusters C8 and C14 comprised over 20% of the cells in AML006 and AML005. These results suggested that although most cells in AML006 and AML005 were chemosensitive and did not match our outcome-predictive subclones, both PDXs included chemoresistant subclones with molecular similarities to our patient-derived outcome-predictive subclones, R1–R5.

Analogously to the analysis of Expanded–Enriched Gene Sets, we sought to identify potential drivers of the differentiation between diminished and transforming subclones. Focusing on the outcome-predictive transforming subclones R3 and R5, we used a pAML-specific regulatory network to identify transcription factors whose inferred activity was dysregulated in R3 and R5 compared to diminished clusters in their corresponding samples; see Methods. Interestingly, our results, summarized in Table S12, identified Retinoid X Receptor Alpha (RXRA) targets as significantly downregulated in R5 relative to the diminished PAUMTZ.d cluster 5 (Figure 5I). The RARA/RXRA complex represses transcription in the absence of retinoic acid^23,24^, and can be activated using all-trans retinoic acid (ATRA) or the agonist tamibarotene^25^. Reanalysis of pAML cell viability in patient samples treated with tamibarotene^26^ revealed a significant negative association between pAML viability and the inferred R5 abundance in these samples based on SQUID deconvolution of their RNA-seq profiles (Figure 5J). Consistent with these observations, SQUID R5 abundance estimates in p401 samples and the pAML cell lines U937 and MOLM13 were significantly reduced after treatment with tamibarotene and ATRA^27^, respectively (Figure 5K). Moreover, patient samples p401 and p198 in Perez et al.^26^ were used to generate PDXs AML001 and AML006, respectively. R5 abundance was high in AML001 but it was not detected in AML006 (Figure 5C and 5G). AML001 demonstrated significant pAML depletion after treatment with tamibarotene, while 50-day surveillance of AML006 suggested that it does not respond to tamibarotene^26^. These results suggested that RARA/RXRA activation specifically reduced the viability of R5 cells.

## DISCUSSION

Little progress has been made toward developing treatment options that improve pAML patient outcomes. Consequently, pediatric oncologists are largely limited to the same chemotherapy agents that were in use 50 years ago, and even after stem cell transplant, more than a third of the patients suffer relapse and die of the disease. Critically, accurate risk prediction at diagnosis and early treatment stages can guide decision-making for how many and which chemotherapy cycles to give and whether to undertake a stem cell transplant after the first remission. The best prognostic indicator currently available is the detection of residual AML cells (i.e., MRD) after one round of chemotherapy^5^, which is associated with a high risk of relapse and poor survival. However, there is no reliable way to discern at diagnosis whether a child will have MRD following the first cycle of chemotherapy, and thus, no way to alter therapy to eradicate this chemoresistant subpopulation. Moreover, nearly 30% of patients with no detected MRD eventually relapse. Given these limitations, we posit that the characterization of chemoresistant cells may reveal the mechanisms by which pAML cells survive cytotoxic chemotherapy and facilitate the development of strategies to disable these mechanisms and improve patient outcomes, thereby achieving a meaningful advance in pAML treatment.

pAML is a heterogeneous, multiclonal disease, and each pAML is composed of genetically and phenotypically distinct subclones with unique gene expression signatures, phosphoprotein signaling responses, and sensitivities to treatment. In this study, we tested the hypothesis that pAML relapse is driven by either chemoresistant pAML subclones that are present before treatment or subclones that transform and gain chemoresistance during treatment. We explored whether similarities between pAML molecular signatures—including high-resolution RNA and protein expression profiles—could be used to identify subclones that are present at both diagnosis and relapse. In addition, we performed longitudinal comparisons of cell profiles before and after treatment to determine if this approach would identify populations that gain chemoresistance. Our results suggest that, indeed, chemoresistant cells are present at diagnosis in over 50% of patients who relapse, and computational inference can help identify pAML subclones that transform from diagnosis to relapse.

In this study, we largely relied on scRNA-seq assays to characterize cell populations in pAML samples. However, our analysis suggested that, although the molecular signatures of tumor-adjacent cell types could be matched across patients, pAMLs are patient-specific. Thus, the integration of pAML cells across patients would likely produce artificially heterogeneous clustering, wherein each cluster comprises a mixture of subclones and cell types with distinct expression profiles. Consequently, we analyzed each patient individually and only performed integration with cells from healthy donors to help indicate adjacent normal cells in each patient sample. Surprisingly, this patient-specific clustering approach revealed clear matches between molecular signatures in diagnosis and relapse samples for more than half of the patients without context-specific computational inference. Similarly, inference of pAML subclones that may have transformed from diagnosis to relapse was possible for the remaining patients using standard scRNA-seq analysis tools.

Crucially, although our findings suggested that expanding and transforming cell clusters represented chemoresistant pAML subclones, additional evidence was needed to support their role as drivers of chemoresistance. To this end, we confirmed the dysregulation of gene sets and pathways that have been associated with pAML chemoresistance and relapse in our candidate subclones. However, classification based on pathway enrichment analysis in bulk pAML profiles was predictive of outcomes for only a small fraction of the patients. This finding may be due to the low abundance of chemoresistant pAML subclones in samples collected at diagnosis—as indicated by analysis of scRNA-seq profiles obtained from these samples—suggesting that the contribution of these subclones to bulk RNA profiles may be too small to detect. To correct this, we employed recently developed methods to predict the abundance of chemoresistant subclones in bulk RNA-seq profiles of pAMLs^6^ and showed that these subclones (R1–R5) were significantly predictive of patient outcomes.

Our approach for identifying pAML subclones that are resistant or gain resistance to chemotherapy lead to dramatically improve prognosis prediction at both diagnosis and during treatment. In particular, we showed that combining pAML subclone abundance—as inferred by SQUID from RNA-seq profiles of diagnostic samples—with cytogenetics-inferred risk and MRD significantly improved pAML outcome prediction (Figures 4I, J). Namely, among the 26% of pAML patients assessed as low risk by all three methods, 90% survived for at least 2 years after diagnosis. In contrast, 12% of pAML patients were assessed as high-risk by all three methods, and only 30% survived for at least 2 years after diagnosis (Table S8). Moreover, tumor composition helped improve risk prediction for both MRD-positive and MRD-negative patients. Most (70%) of the MRD-positive pAML patients had either high-risk cytogenetics or high abundance of poor outcome–predictive subclones (T), and these patients had worse survival (31% survival for at least 2 years) than other (30%) MRD-positive pAML patients (64% survival). Analogously, 37% of the MRD-negative patients were identified as high-risk by both cytogenetics and T, and 43% of these patients died of disease, compared to only 16% of the other MRD-negative patients. Thus, our findings suggest that the combination of the above three prognostic features can be leveraged to identify most pAML patients who will relapse after a complete response to chemotherapy, as well as those who will only partially respond to chemotherapy and should be offered alternative therapies.

We further evaluated our chemoresistant subclones *in vivo* using PDX models that are hypothesized to faithfully represent both chemosensitive and chemoresistant pAMLs. Our analysis revealed that PDX models recapitulate pAML heterogeneity, and pAML PDX subclone response to chemotherapy can be used to evaluate observed phenotypes. Indeed, even chemoresistant PDXs were composed of multiple pAML subclones with distinct molecular signatures. Importantly, PDX models that responded to chemotherapy comprised both chemosensitive and chemoresistant subclones, which diminished and expanded, respectively, following treatment; note that our models were derived from pre-treatment samples from relapsed patients. Moreover, all tested PDXs had cells that closely resembled the signatures of patient pAML subclones that expanded or transformed and were predictive of poor patient outcomes (T); see Figure 5C. However, these cells were at markedly lower abundance in chemosensitive PDXs. A higher resolution analysis revealed that T matched the molecular signatures of chemoresistant PDX subclones—but not of chemosensitive subclones—in both chemosensitive and chemoresistant PDXs. This analysis suggested that the high variability in PDX response to chemotherapy may be driven by the abundance of their chemoresistant PDX subclones, and further supported our hypothesis that pAML therapeutic response is driven by individual subclones exhibiting differing responses to treatment, even when the overall tumors seemingly respond to the therapy.

Notably, the identification of outcome-predictive chemoresistant subclones confirmed the relevance of key pAML pathways and further identified new targetable pathways associated with chemoresistant pAML. Namely, the activation of MYC^1^ and the oxidative phosphorylation pathway^10^ have been previously implicated with chemoresistance in pAML, as was the differentiation state of pAML and corresponding stem cell signatures LSC47 and HSC^3,16^, all of which were identified in our analyses. Furthermore, despite our focus on predicting outcomes of FLT3-ITD–negative patients, the most enriched gene sets in outcome-predictive pAML subclones and PDXs—none of which were derived from pAMLs with FLT3-ITD mutations—were associated with FLT3/CDK6 upregulation. However, as previously observed for LSC47 and HSC, efforts to predict outcomes and responses to therapies based on the enrichment of these gene sets failed for most patients. Our analysis suggests that these results are due to pAML heterogeneity, with chemoresistant subclones potentially accounting for less than 10% of the pAML cell population at diagnosis.

We argued for therapies that specifically target chemoresistant pAML subclones. These include targeting the FLT3/CDK6 pathway when select pAML subclones are detected even in the absence of FLT3 ITDs, and we specifically evaluated the use of retinoic acid receptor agonists for tumors that contain cells resembling the R5 subclone. We showed that patient samples, cell lines, and PDXs with high R5 composition can be effectively targeted by retinoic acid receptor agonists, while samples and PDXs with low R5 composition failed to respond to these treatments. We note that all-trans retinoic acid and tamibarotene are actively being studied in phase 2 and 3 clinical trials as therapies for adult AML patients who are unlikely to tolerate standard intensive chemotherapy. In conclusion, our study underscores the power of combining healthy donor samples, paired longitudinal pAML profiles, a large cohort with bulk expression data and clinical annotation, faithful animal models, and accurate deconvolution methods to enable the identification of outcome-predictive subclones that can be applied to improve prognosis-prediction methods for pAML patients. Our analyses of these integrated data proposed improved diagnostic and personalized therapies for pAML patients.

## METHODS

### Patient samples

Bone marrow or blood samples were collected at diagnosis and relapse for pAML patients whose families consented to banking tissue for research following the Declaration of Helsinki. Six diagnosis–relapse pairs were obtained from patients diagnosed and treated at TCH. Nine diagnosis–relapse pairs were obtained from the COG Biopathology Center. All patients received initial chemotherapy while enrolled in or according to the protocol used in the Phase 3 COG clinical trial AAML1031^11^. Demographic and clinical information are listed in Supplemental Table S1. Two normal bone marrow samples were obtained from healthy donors by rinsing the discarded collection filter. All COG samples were enriched for mononuclear cells by density centrifugation and cryopreserved; these samples were subjected to fluorescence-activated cell sorting (FACS) to enrich for AML cells (side scatter [SSC] low, CD45 dim) prior to downstream analyses. Samples from the TCH cases were sent for single cell sequencing without prior sorting. To control for the effects of sorting, one normal bone marrow aspirate was split in two, and only half the cells were FACS sorted to enrich for immature myeloid cells in the same gate as used for the COG cases. All six TCH cases and seven of the nine COG cases passed quality control (QC) for inclusion in our analyses.

### scRNA-seq profiling and analysis

#### Profiling.

Patient samples were flow-sorted, gating for CD45-dim and SSC low cells; 10K–15K cells were then labeled using the 10x Genomics (Pleasanton, CA) Chromium Next GEM Single Cell 3’ Kit v3.1 and sequenced at 200 M reads per sample using an Illumina NovaSeq 6000. The resulting scRNA-seq data were analyzed by an anchor-based approach with the *Seurat* package v4.30. Specifically, cells were analyzed using three distinct methods. First, cell counts were log normalized and clustered using the Louvain algorithm. Cells across all profiled and collected samples were then merged (Figure 1) using standard *Seurat* v4.30 packages^13^. Second, cell profiles from paired samples and normal samples were normalized and integrated one patient at a time to classify pAML and non-tumor cells (Figure 2). Finally, patient-specific cell clusters were combined to merge similar cells within and across patients and reduce the cell space (Figure 4).

#### Cell classification.

To analyze patient-specific cell populations, we first log normalized the count data from each patient pair containing diagnosis and relapse samples together with data from three normal bone marrow samples from two individuals (TCHnorm1 and TCHnorm2). Normalized data were then integrated by their sample types (tumor, normal) using the *IntegrateData* function, and cell clustering was performed on the integrated data using the Louvain algorithm at a resolution of 2.5. We identified *expanded subclones* as integrated cell clusters comprising cells from both the diagnosis and relapse samples in which >10% of the cells from the relapse sample but not from the diagnosis sample passed QC. Similarly, *diminished subclones* were identified as clusters for which >10% of the cells from the diagnosis sample but not the relapse sample passed QC, whereas *stable subclones* were classified as those for which >10% of cells from both paired samples passed QC. Clusters that included >10% of the cells from the relapse sample were identified as *relapsed subclones*. The remaining clusters, including small clusters or clusters that were mostly composed of non-pAML cells, were identified as *other cells*. When combining clusters across patients, we merged clusters with similar (psuedobulk) expression profiles and argued, based on parameters set by SQUID, that such clusters are derived from the same pAML subclones.

#### Deconvolution.

We used the SQUID framework to predict the abundance of scRNA-seq clusters and subclones in bulk RNA-seq profiled samples. The first step of SQUID’s framework is the combination of cell clusters with similar expression patterns. This is accomplished by first estimating patient-specific cell population similarity by performing pairwise Pearson correlations of the mean log-normalized expression profile of each cluster, then, high-similarity groups that included at least two clusters with r>0.986 were combined to produce an aggregate cluster. Note that the number of effective expression profiles defining cluster centroids under this formulation is many magnitudes greater than the number of cell types in the dataset. Consequently, cell populations with highly similar profiles are expected to be homogenous, which allows molecular signatures from scRNA-seq profiles to indicate cell identities. This merging operation and the disqualification of clusters with fewer than 50 cells reduced the cell-type space from 138 patient-specific clusters to 90 clusters (pAML subclones and normal adjacent cell types). These clusters were used to deconvolve RNA-seq profiles of patient samples, PDXs, and cell lines.

#### Cell type inference.

We used the Celldex package v.2.0.0 in SingleR^15^ to determine the cell type of each population. However, to enable higher-resolution cell classification, including the distinction between multiple myeloid cell differentiation stages, we combined reference databases, including the Human Primary Cell Atlas^28^, Novershtern’s Hematopoietic Data^29^, Blueprint and ENCODE^30^, the Database of Immune Cell Expression^31^, and Monaco’s Sorted Human Immune Cells Database^32^.

#### Trajectory analysis.

We performed single-cell trajectory analysis with the monocle3 package v1.3.1^33^ to infer potential diagnosis-to-relapse transformation trajectories and identified cells in diagnostic samples that may transform during treatment. The data were normalized using the *size_only* method, and data dimensionality was reduced to 100 with pseudocount=0. A principal graph was fitted using the *learn_graph* function to construct the trajectory path.

#### PDX-profile resolution.

When analyzing bulk RNA-seq and scRNA-seq profiles of PDX samples, we classified the xenograft-derived sequence read data using *Xenome*^34^, which effectively handles a mix of reads from both the host and the graft, for further analyses.

### CyTOF

Five COG diagnosis–relapse pairs profiled by scRNA-seq had a sufficient number of cells to perform CyTOF. After sorting, cells allocated for CyTOF were divided into two fractions; one was stimulated for 15 min with conditioned medium from HS-5 bone marrow stromal cells to activate intracellular signaling^35^, and the other was left unstimulated. Cells were fixed in 1.6% paraformaldehyde and barcoded (Cell-ID^™^ 20-Plex Pd Barcoding Kit, Fluidigm) for multiplexing, which allows combining cells for CyTOF analysis to reduce batch effects. Samples were then stained with 38 surface marker antibodies, permeabilized, and stained again for eight intracellular antigens; see Table S11 for the antibodies used. Normalization beads were included with every run. Doublets and non-viable (caspase 7^+^) cells were excluded. CyTOF assays were performed by the Baylor Cytometry and Cell Sorting Core. The data were compiled, cleaned, gated, and analyzed in Cytobank. Dimensionality reduction with UMAP was performed for each diagnosis–relapse pair using all surface and intracellular markers, except for cleaved caspase 7, which was used for the exclusion of non-viable cells.

### GSEA to identify Expanded–Enriched Gene Sets and drivers of pAML transformation

Expanded Genes were identified by differential expression analysis comparing each expanded, relapsed, and stable cluster to all diminished clusters in each patient (separately) using Wilcoxon Rank–Sum tests. Genes expressed in at least 25% of the cells with an adjusted p<0.05 were considered significant for this comparison. Expanded Genes were further selected as those significantly upregulated in at least 21 out of the 52 comparisons made, selecting this cutoff because it splits the bimodal distribution of tallies across all candidate genes. We then used *enrichr*^36^ or hypeR^37^ with adjusted p<0.05 to identify gene sets and pathways that were enriched among Expanded Genes. We reverse-engineered a pAML-specific regulatory network based on TARGET pAML gene expression profiles as previously described^38–40^. This network was used to identify pAML-specific transcription factor (TF) targets that were used as gene sets for GSEA analysis. This, in turn, was used to estimate TF activity when comparing expression profiles across cell populations.

### Evaluating the predictive ability of models and gene sets in TARGET and St. Jude pAML datasets

We used pAML clinical characterizations and RNA-seq profiles collected by TARGET (1,435 samples) and St. Jude Research Hospital (170 samples) to evaluate the predictive ability of gene sets and models. In total, TARGET included 1,330 diagnosis, 43 relapse, and 62 healthy donor bone marrow samples, whereas the St. Jude collection included 37 diagnosis and 133 relapse samples. RNA-seq count data were log normalized, and we performed GSVA^41^ analysis to evaluate whether Expanded–Enriched Gene Sets were upregulated in diagnosis and relapse samples (Figures 3D, 3E, and S5). Comparisons between samples from healthy donors, pAML diagnosis, and pAML relapse samples were evaluated by Wilcoxon Rank–Sum tests. The same GSVA scores were also used to evaluate the predictive ability gene sets for patient outcomes (Figures S6–S10).

We next used SQUID^6^ to evaluate the abundance of 90 patient-specific clusters, including non-pAML cells, in TARGET diagnosis samples (reduced from 138 clusters after merging similar clusters and eliminating poorly characterized clusters). Among these 90 clusters, 49 were expanded, 3 were stable clusters, and 14 were transforming-cell clusters. Clusters were evaluated for predictive ability for both OS and EFS by Cox regression with Bonferroni corrections for multiple testing. Outcome-predictive models (R1–R5) were composed of diagnosis sample cells and were predictive of both OS and EFS at an adjusted p<0.01. Tests of other clusters yielded none that were outcome predictive. Because FLT3-ITD-positive patients were treated differently on the study’s protocol, we focused our analysis on the 870 FLT3–ITD–negative patients with profiled diagnosis samples.

In survival analyses (Figures 4B–G), patients were equally partitioned based on the median inferred subclone abundance. Survival analysis based on MRD after one or two rounds of chemotherapy split patients into MRD-positive or unknown vs. MRD-negative groups, and analysis based on cytogenetics assigned patients with MLL rearrangements or other alterations to the high-risk category and all other patients to the low-risk category (Figure S12). Feature combinations partitioned the patient populations into discrete groups using binary features to represent total outcome-predictive cluster abundance (T), MRD, and cytogenetics (Figures 5I–K). Higher-resolution analyses of cytogenetics biomarkers are provided in Figures S12 and S13. In addition, we performed analyses using MRD after two rounds of chemotherapy and the AAML1831 risk-prediction algorithm (Figure S13, NCT04293562). However, these variations did not alter the conclusions of our presented analysis based on TARGET-provided cytogenetics biomarker aggregations and MRD alone; note that including MRD after two rounds of chemotherapy did not improve predictive accuracy, and the AAML1831 risk-prediction algorithm is a refinement of the classic cytogenetics classification. A multivariate Cox proportional hazard model assessing T, cytogenetics, MRD, and these factors combined was further constructed using the *coxph* function in R. The p-value associated with each category or sub-category within the model is reported in Figure S14B. For categorical variables, normal cytogenetics and MRD-negative were used as the reference levels for modeling cytogenetics and MRD, respectively.

### PDX experiments and analysis

Established serially transplanting pediatric AML PDX models from the PALeX resource were used for the experiments reported in this study (https://pdxportal.research.bcm.edu/pdxportal)^42^. Characteristics of patients from whom the models were derived are listed in Supplemental Table S9. For each experiment, 6–10 immunodeficient, human cytokine–producing mice (NSGS, MISTRG, or MISTRG6) were injected by tail vein with 2×10^5^ viable AML cells that were harvested from a prior passage and cryopreserved. Once mice demonstrated at least 2% human AML (hAML; human CD45^+^/CD3^−^) cells in peripheral blood, they were assigned to receive cytarabine or saline (Figure S15). Treatment groups were balanced for peripheral blood disease burden. Cytarabine (50 mg/kg) was administered via intraperitoneal injection once daily for 4 days; control animals received an equal volume of saline. All mice were humanely euthanized on day 4 following the last dose of cytarabine or saline control. Bone marrow and spleen were harvested, and the percent hAML was determined by flow cytometry. Bone marrow from one cytarabine-treated and one saline-treated mouse was cryopreserved and sent for profiling by RNA-seq and scRNA-seq. We then used SQUID to evaluate the response-predictive ability of our models based on bulk RNA-seq–profiled PDXs, following the same protocol as that employed for TARGET pAML samples—using the 90 clusters derived from scRNA-seq profiles of our 13 pAMLs with paired diagnosis–relapse samples. We further used diagonally weighted least squares estimation^43^ to compare the outcome-predictive clusters R1–R5 with PDX subclones and GSVA to evaluate the enrichment of Expanded–Enriched Gene Sets in PDX subclone pseudobulk profiles. The effects of 1 μM treatment with all-trans retinoic acid (ATRA) in U937 and MOLM13 at 72 hours were estimated by Meier et al. with genes profiled by RNA-seq^27^. The effects of patient sample treatment by tamibarotene (100nM) at 24 hours were estimated by Perez et al. with genes profiled by RNA-seq^26^. We used SQUID to estimate the abundance of R5 in these samples, deconvolving RNA-seq profiles using all 90 clusters as input. We note that the PDXs AML001 and AML006 were derived from patient samples p401 and p198, respectively, in Perez et al.

## Supplementary Material

Supplement 1

Supplementary Files

This is a list of supplementary files associated with this preprint. Click to download.

• TableS1PatientsClinicalData.xlsx

• TableS7ExpandedGeneEnrichedSignatures.xlsx

• TableS2ProportionalTableMetaClustersFig1C.xlsx

• TableS4CombinedCellTypevsClustertables.xlsx

• TableS3CombinedCellClustersproportiontables.xlsx

• TableS11cytofantibodypanels.xlsx

• TableS9MultivariateCoxanalysisofRs.xlsx

• TableS6MapFig1toFig4AClusters.xlsx

• TableS10PDXmodels.xlsx

• TableS8DOD.xlsx

• TableS12TFactivityinTransformingCells.xlsx

• TableS5DEGsincomparingexpandedvsdiminishedclusters.xlsx

## Data Availability

TCH pAML scRNA-seq and bulk RNA-seq datasets as well as the COG pAML scRNA-seq dataset are available at GEO Super Series GSE271137. The COG pAML CyTOF profile data reported in this paper are publicly available via Cyto-bank.
