# Supplementary material for: Pediatric acute myeloid leukemia tumor composition predicts patient outcomes at diagnosis and reveals mechanisms of resistance to chemotherapy
Source: Res Sq. 2026 Jan 23:rs.3.rs-4669225. Preprint. [Version 1] doi: 10.21203/rs.3.rs-4669225/v1 (PMC12869664; doi:10.21203/rs.3.rs-4669225/v1)
Supplement: Supplement 1 [file NIHPPrs4669225v1-supplement-1.pdf]

SUPPLEMENTARY FIGURES

Figure S1. Uniform manifold approximation and projection (UMAP) visualization of single-cell RNA sequencing expression profiles illustrates the distribution of single cells across datasets of pediatric acute myeloid leukemia (pAML) patients and in bone marrow samples from non-cancer donors (indicated by a black line).

Figure S2. UMAP visualizations of cell clusters in the 13 paired diagnosis–relapse samples after integrated analysis of each patient's diagnosis and relapse samples with three non-cancer bone marrow donor controls. Cell clusters (top) and time points (bottom) for each of the 13 sample pairs are indicated.

Figure S3. Our proposed model for the cell composition of pAML patient samples at diagnosis and relapse accounts for tumor-adjacent cells and both chemoresistant and chemosensitive cells in the diagnosis sample. This model proposes the following: **1**) the relative proportion of tumor-adjacent cells in the sample may increase or decrease between diagnosis and relapse, **2**) the relative proportion of chemoresistant cells at diagnosis (expanded cells) will increase during treatment, and **3**) the relative proportion of some chemosensitive cells will diminish (diminished cells), whereas others may transform and gain resistance to chemotherapy. We aim to characterize expanded and transforming cells that are predictive of pAML patient outcomes.

Figure S4. (A, B) Single-cell trajectory plots depict representative samples from patients with or without expanding cells in diagnosis samples (i.e., Group A and Group B patients, respectively). In the top panel, diagnosis and relapse time points are color-coded in red and blue, respectively, with the trajectory path indicated by a solid black line. In the bottom panel, clusters are categorized as expanded (in light red), diminished (in light blue), or other (in gray), with the trajectory path represented by a solid yellow line. Note that for Group A samples, subclones are present in both the diagnosis and relapse samples, while for Group B samples, samples are distinct. However, the trajectory path predicts cell transformation for subclones from both groups. **(C)** A comparison of the time to relapse for patients in Group A vs. Group B.

Figure S5. (A) Gene Set Variation Analysis (GSVA) scores of FLT3–co-expressed genes in our Expanded–Enriched Gene Sets were significantly higher for FLT3-ITD–positive than FLT3-ITD–negative Therapeutically Applicable Research to Generate Effective Treatments (TARGET) diagnostic profiles. (**B**) FLT3, cyclin-dependent kinase 6 (CDK6), Expanded Genes, and the leukemia stem cell populations gene set (LSC47) are upregulated in FLT3-ITD–positive TARGET diagnostic profiles.

Figure S6–S10. Survival analysis based on GSVA scores of our Expanded–Enriched Gene Sets in TARGET-profiled AAML1031 FLT3-ITD–positive pAMLs, FLT3-ITD–negative pAMLs, FLT3-ITD–negative mixed-lineage leukemia (MLL)-rearranged pAMLs, FLT3-ITD–negative pAMLs with inversion 16 (*CBFB::MYH11*), and FLT3-ITD–negative pAMLs with *RUNX1::RUNX1T1* translocations.

Figure S11. Event-free survival of TARGET-profiled FLT3-ITD–negative pAML patients as a function of the abundance of our outcome-predictive pAML subclones (R1–R5) and their total abundance (T); analogous to Figure 4B–G.

Figure S12. Survival analysis based on TARGET profiles of FLT3-ITD–negative pAMLs using (**A**) standard cytogenetics biomarkers included in TARGET, (**B**) the AAML1831 risk-prediction algorithm, and (**C**) measurable residual disease (MRD) after one round of chemotherapy.

Figure S13. Survival analysis based on TARGET profiles of FLT3-ITD–negative pAMLs using a combination of the total abundance of our outcome-predictive pAML subclones (T) and (**A**) standard

cytogenetics biomarkers included in TARGET, (**B**) the AAML1831 risk-prediction algorithm, and (**C**) MRD after one and two rounds of chemotherapy. (**D**) Analogously, event-free survival of patients categorized based on T, MRD after one round, and MRD after two rounds of chemotherapy.

Figure S14. (A) Quantile analysis of regression using outcome-predictive features, standard cytogenetics biomarkers included in TARGET, MRD after one round of chemotherapy, and the combination of these features suggests a significantly higher accuracy for outcome prediction with the combined feature set. (**B**) Evaluation of a multivariate Cox proportional hazard model for T, cytogenetics, and MRD using the *coxph* function in R. For categorical variables, normal cytogenetics and MRD negativity were set as the reference levels for modeling cytogenetics and MRD, respectively. The MRD-unknown category likely includes patients who were too sick to undergo bone marrow aspiration. T, the inferred total abundance of subclones R1–R5, is a continuous variable, and we present the hazard ratio associated with median T in TARGET samples. Observed maximum T, with 40% of cells matching R1–R5, is associated with a 3.5 hazard ratio.

Figure S15. (A) Pre-treatment $CD45^+$ cell abundance in PDX samples was used to determine treatment initiation time point. (**B**) Graphs showing the number of $CD34^+CD38^-$ and $CD34^+CD38^+$ pAML cells among total residual human $CD45^+CD33^+$ cells identified in patient-derived xenograft (PDX) models AML006, AML005, AML001, and AML010 after treatment with cytarabine (AraC; red bars) or saline (blue bars. (**C**) Total $CD45^+CD33^+$, $CD34^+CD38^-$, and $CD34^+CD38^+$ pAML cells in PDX AML006 after treatment with AraC (red bars) or saline (blue bars).

SUPPLEMENTARY TABLES

Table S1. Clinical annotation of each profiled pediatric acute myeloid leukemia (pAML) patient, including age, sex, cytogenetics, mutations, time to relapse, measurable residual disease (MRD), and sample blast abundance.

Table S2. The table form of Figure 1C shows cluster frequencies in each profiled sample.

Table S3. Cluster and subclone frequency tables corresponding to Figure 2A for each analyzed patient. Cell counts by sample are provided. Each tab provides cell counts per cluster where the profiles of a patient sample and 3 normal bone marrow samples were merged.

Table S4. Total cell counts and predicted cell type for each cluster that was reported in Table S3.

Table S5. Differentially expressed genes and the statistics used for each comparison, including comparisons reported in Figures 3A, B.

Table S6. Map of the merging operation used to reduce the set of clusters reported in Tables S3 and S4 to a total of 90 clusters (pAML subclones and normal-adjacent cell types). These 90 clusters were used to predict pAML subclone abundance in Figures 4 and 5. Maps to individual samples and between the UMAPs reported in Figures 1 and 3 are also provided. Cell counts per cluster are provided.

Table S7. The identity of our Expanded–Enriched Gene Signatures.

Table S8. Therapeutically Applicable Research to Generate Effective Treatments (TARGET)-profiled patient overall survival data as classified by our three predictive features.

Table S9. Multivariate Cox analysis of our outcome-predictive pAML cell abundance, classic cytogenetics biomarkers evaluated by TARGET, and MRD.

Table S10. Annotation of the patient-derived xenograft (PDXs) used in this study, including information about donor pAML patients and pre-treatment $CD45^+$ PDX cell abundance.

Table S11. The antibodies that were used to analyze the five pAML profiles using cytometry by time of flight (CyTOF). Both raw and normalized data were deposited in Cytobank.

Table S12. Inferred transcription factor activity (by GSEA) in the two outcome-predictive transforming cell clusters, when compared to diminishing cells in the same sample; p values were Bonferroni corrected.

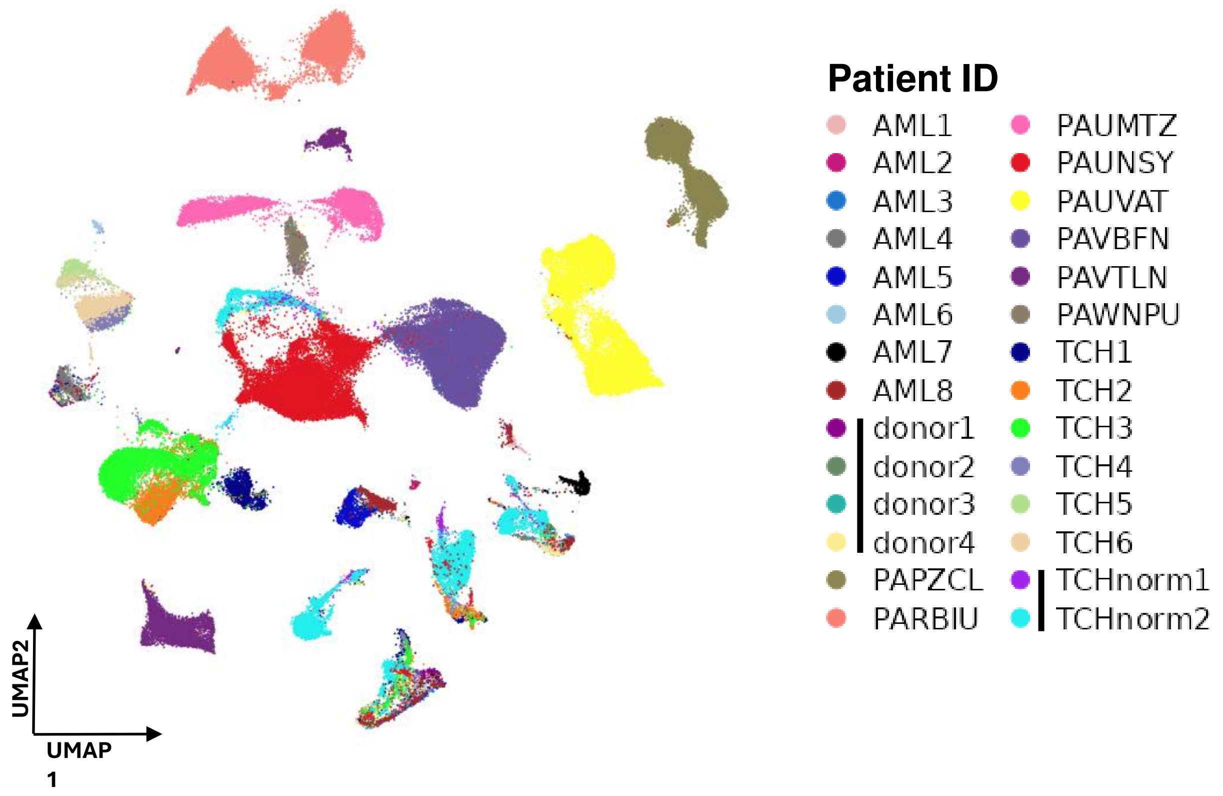
Patient ID
AML1
AML2
AML3
AML4
AML5
AML6
AML7
AML8
donor1
donor2
donor3
donor4
PAPZCL
PARBIU
PAUMTZ
PAUNSY
PAUVAT
PAVBFN
PAVTLN
PAWNPU
TCH1
TCH2
TCH3
TCH4
TCH5
TCH6
TCHnorm1
TCHnorm2
UMAP2
UMAP 1

Figure S1

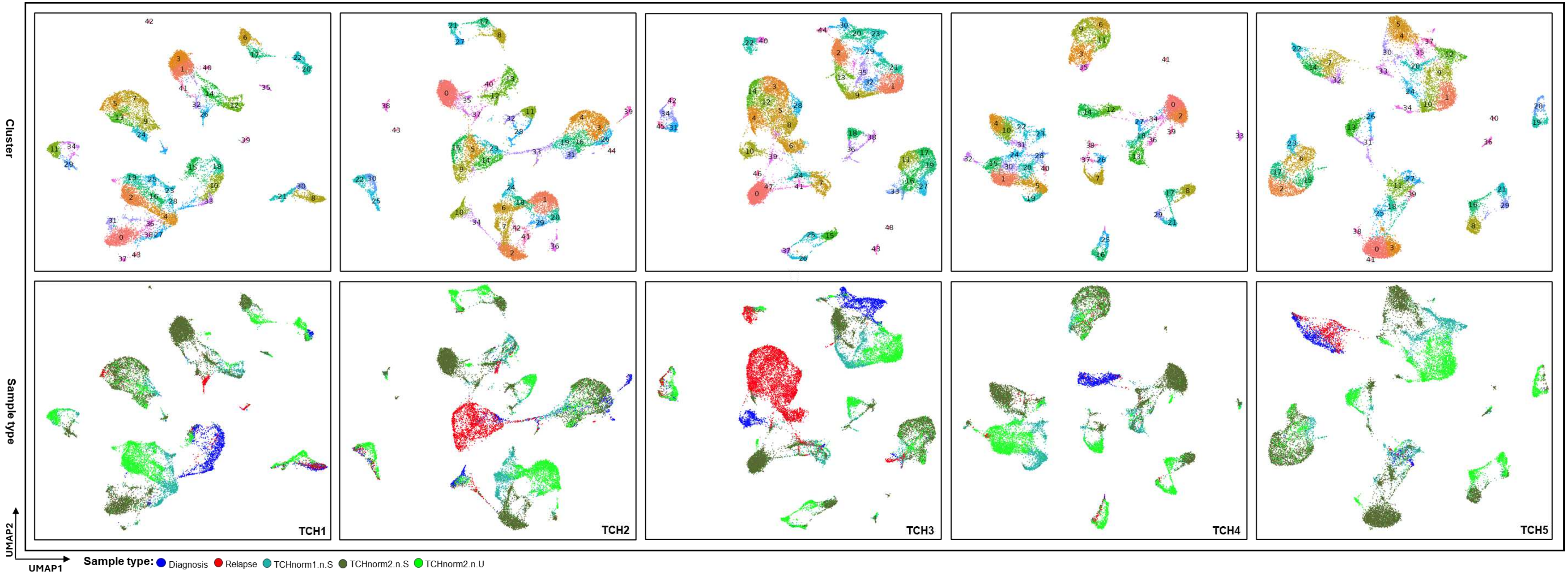
Cluster
Sample type
TCH1
TCH2
TCH3
TCH4
TCH5
UMAP2
UMAP1
Sample type: Diagnosis Relapse TCHnorm1.n.S TCHnorm2.n.S TCHnorm2.n.U

Figure S2

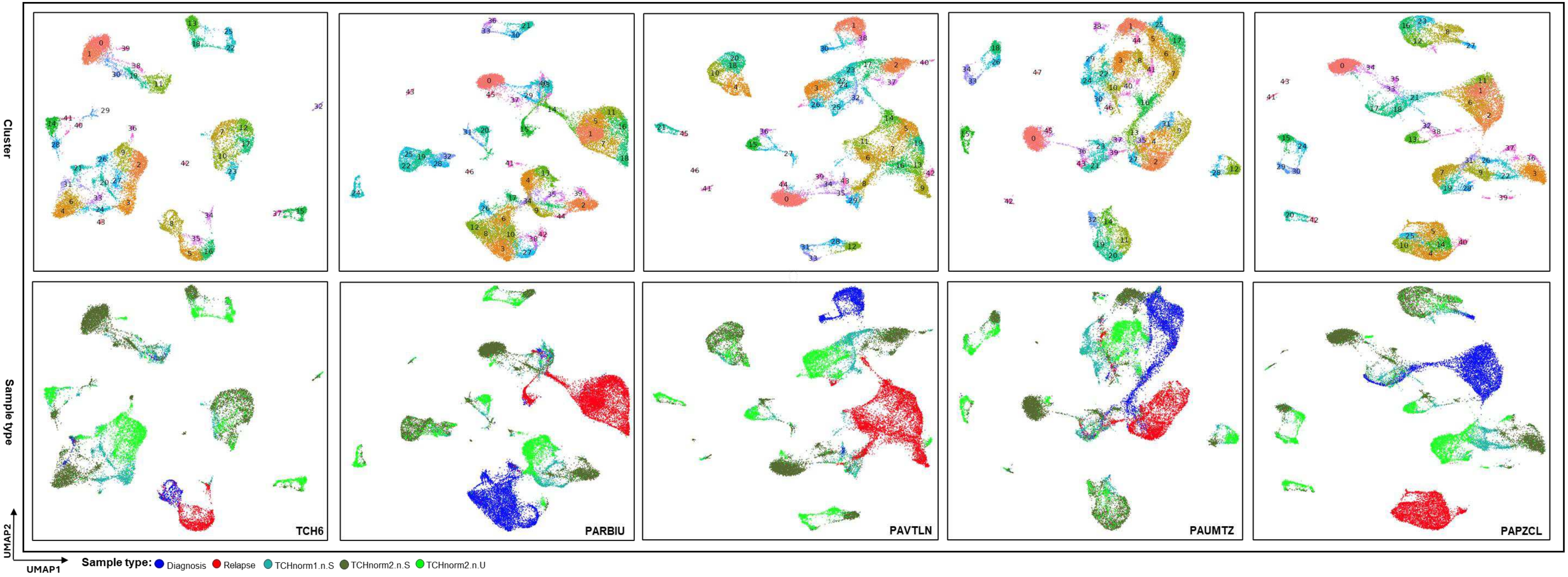

Figure S2 (Continued)

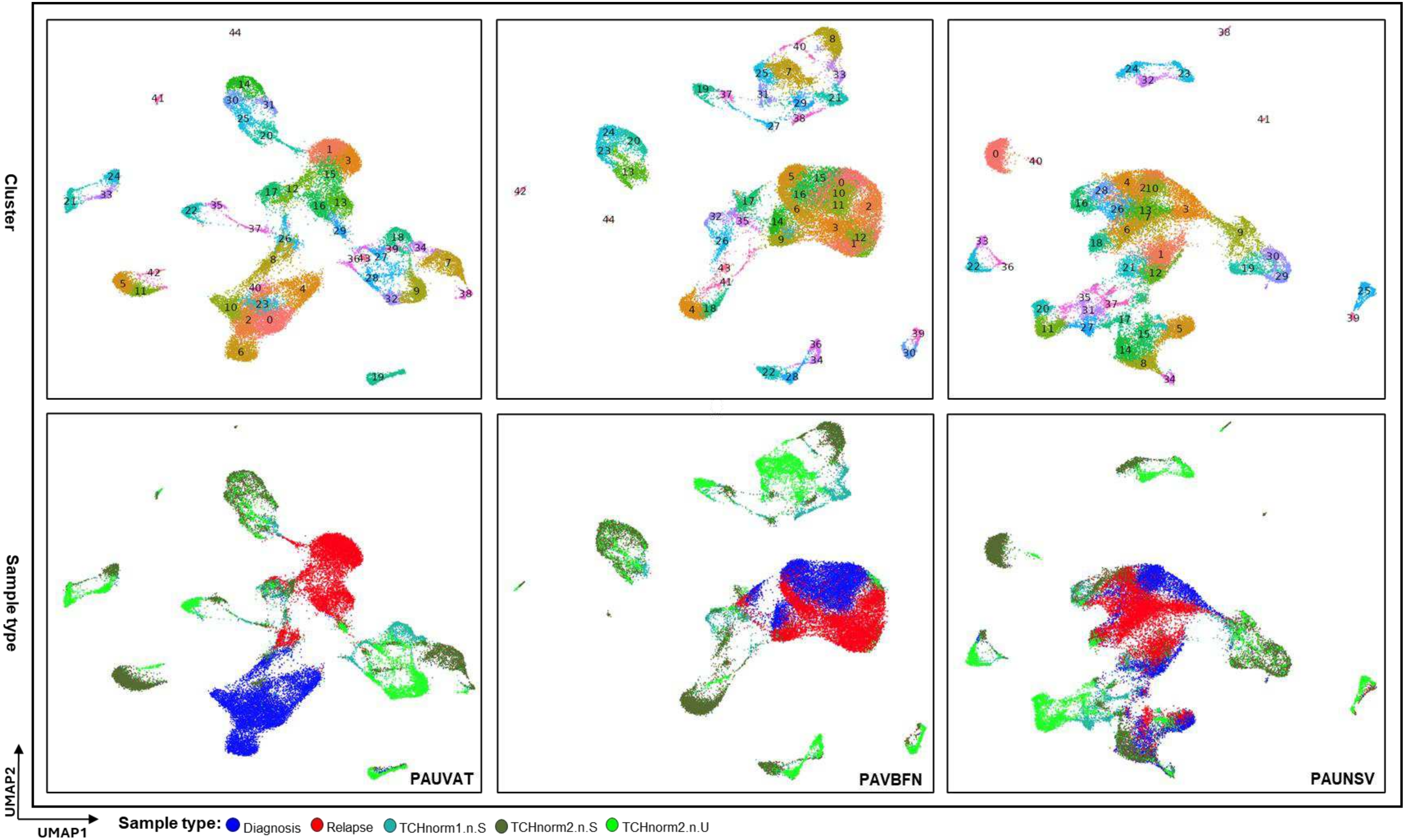

Figure S2 (Continued)

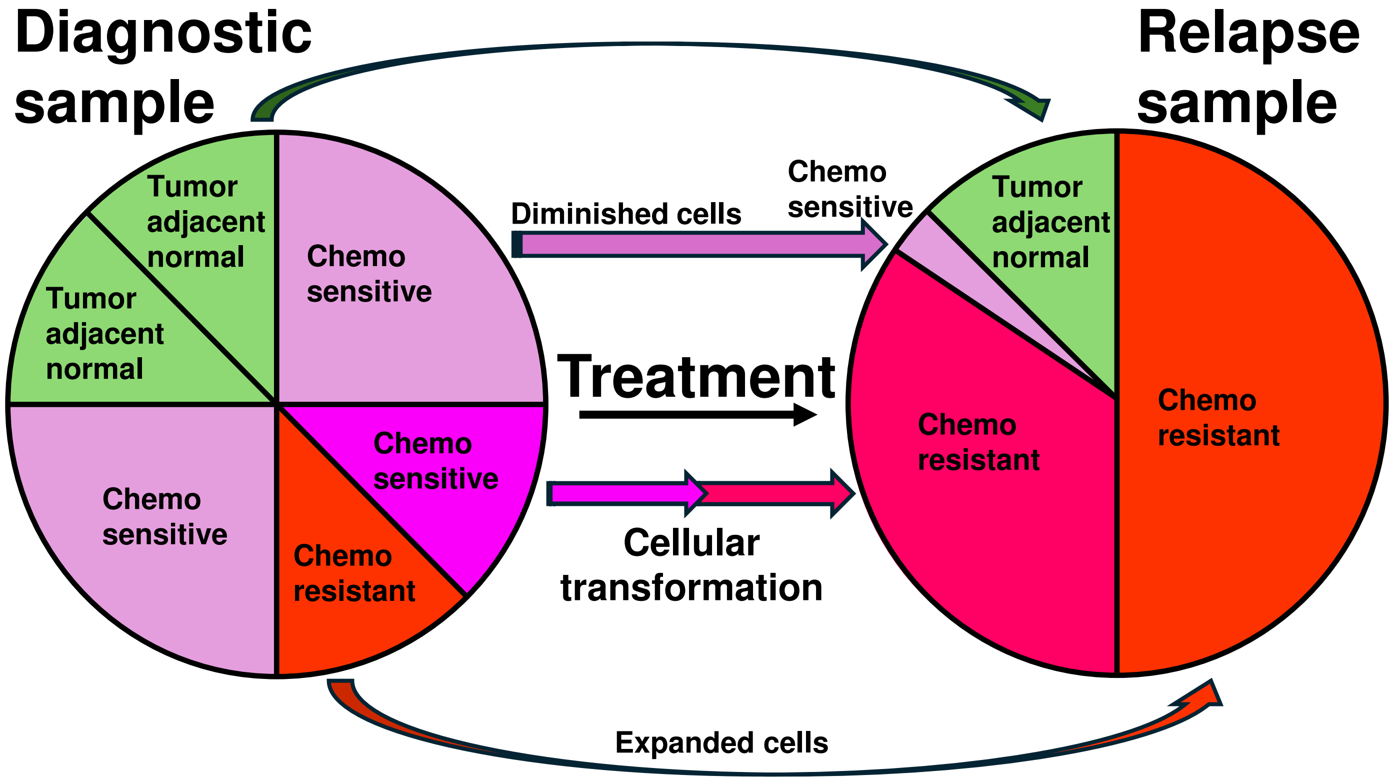
Diagnostic sample
Relapse sample
Tumor adjacent normal
Tumor adjacent normal
Chemo sensitive
Chemo sensitive
Chemo sensitive
Chemo resistant
Diminished cells
Chemo sensitive
Treatment
Cellular transformation
Tumor adjacent normal
Chemo resistant
Chemo resistant
Expanded cells

Figure S3

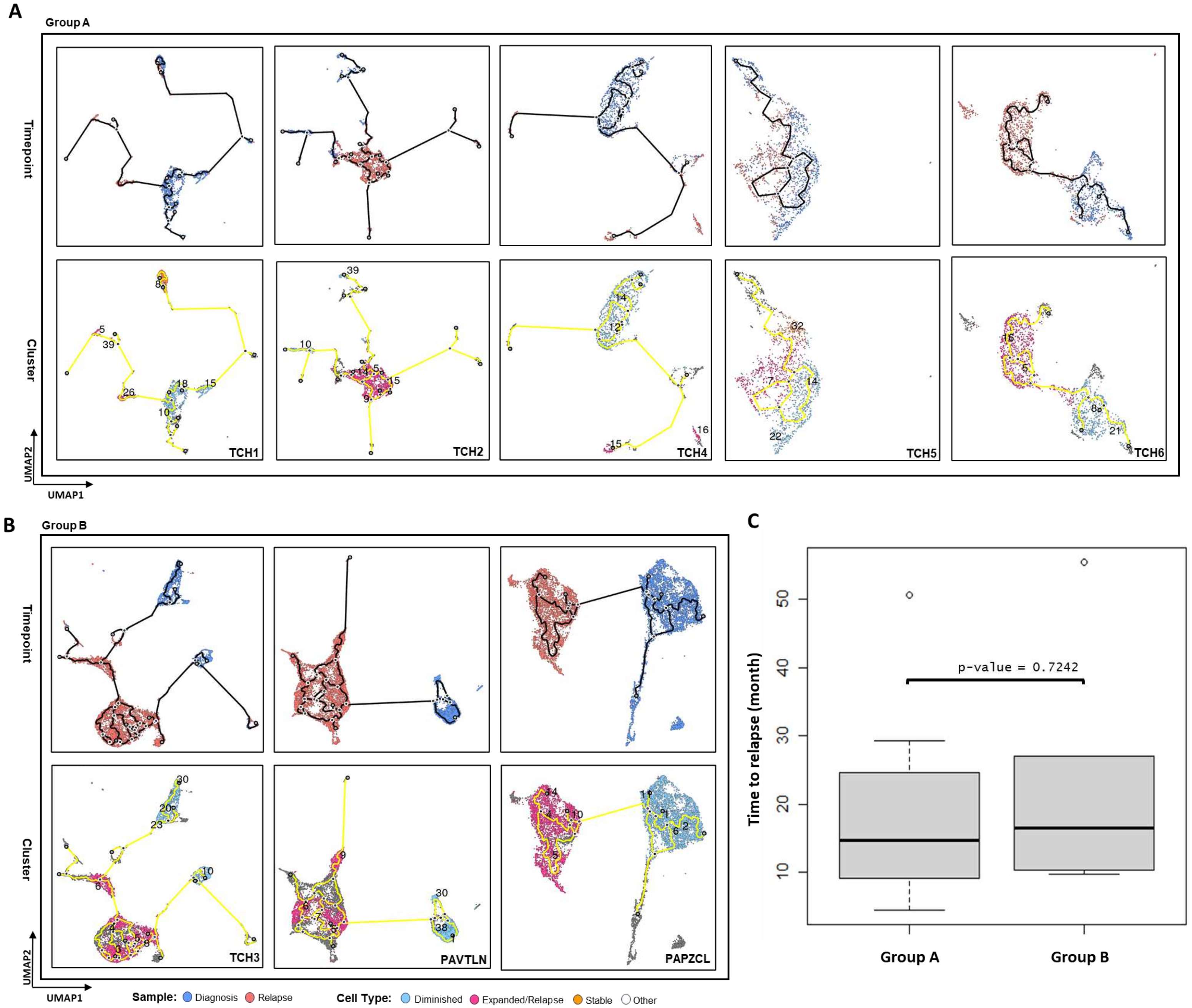
A
Group A
Timepoint
Cluster
TCH1
TCH2
TCH4
TCH5
TCH6
UMAP2
UMAP1
B
Group B
Timepoint
Cluster
TCH3
PAVTLN
PAPZCL
UMAP2
UMAP1
Sample: Diagnosis Relapse
Cell Type: Diminished Expanded/Relapse Stable Other
C
Time to relapse (month)
p-value = 0.7242
Group A
Group B

Figure S4

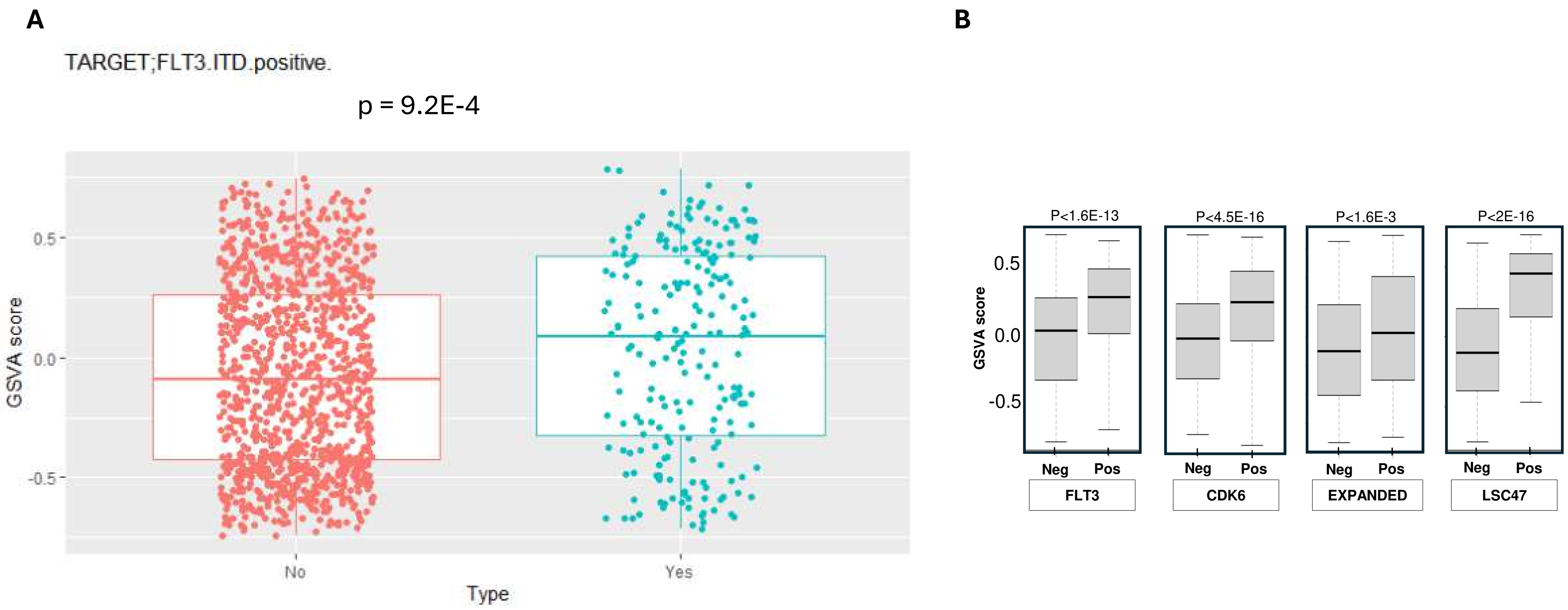
A
TARGET;FLT3.ITD.positive.
p = 9.2E-4
GSVA score
0.5
0.0
-0.5
No
Yes
Type
B
P<1.6E-13
P<4.5E-16
P<1.6E-3
P<2E-16
GSVA score
0.5
0.0
-0.5
Neg
Pos
FLT3
CDK6
EXPANDED
LSC47

Figure S5

AAML1031 FLT3-ITD Positive (n=169)

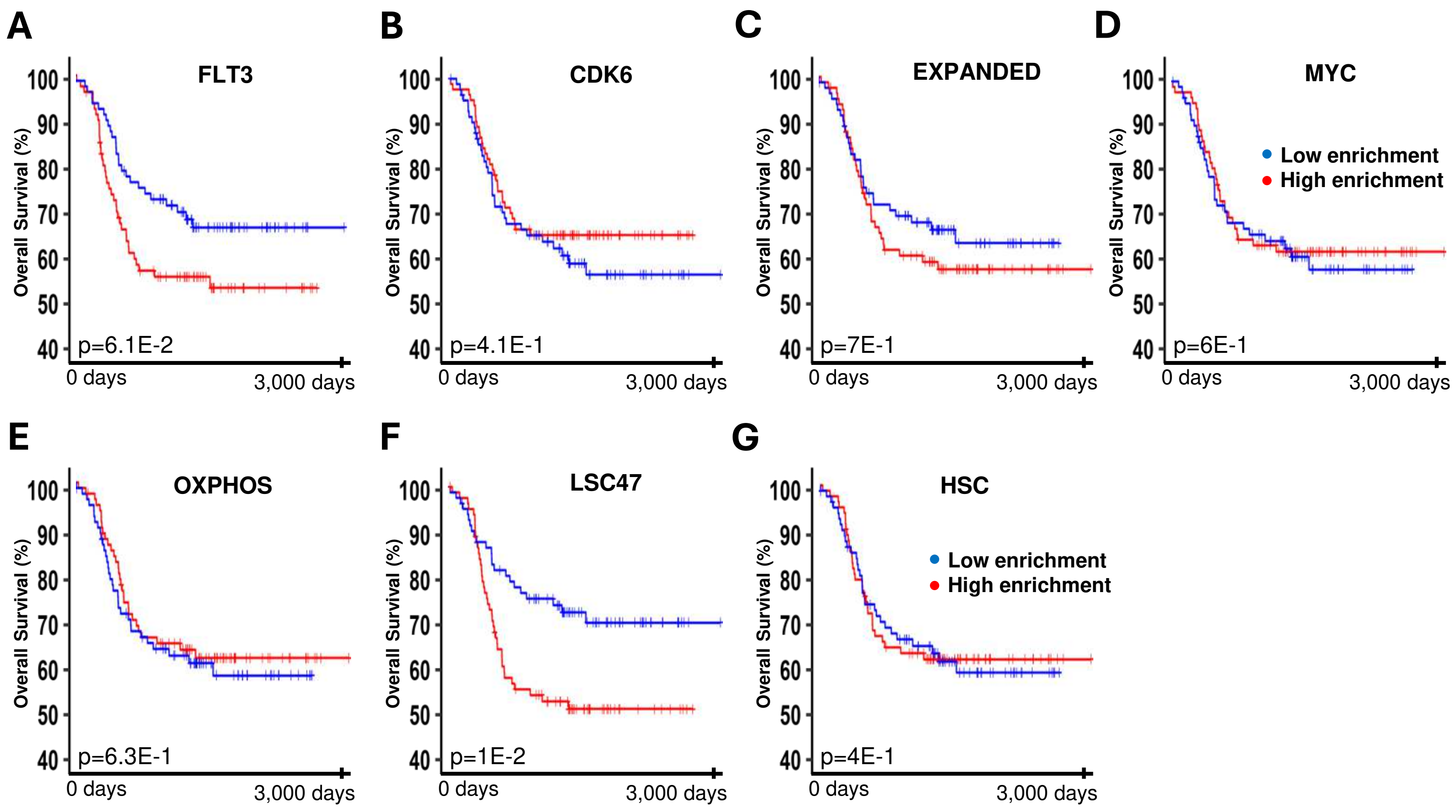

Figure S6

AAML1031 FLT3-ITD Negative (n=870)

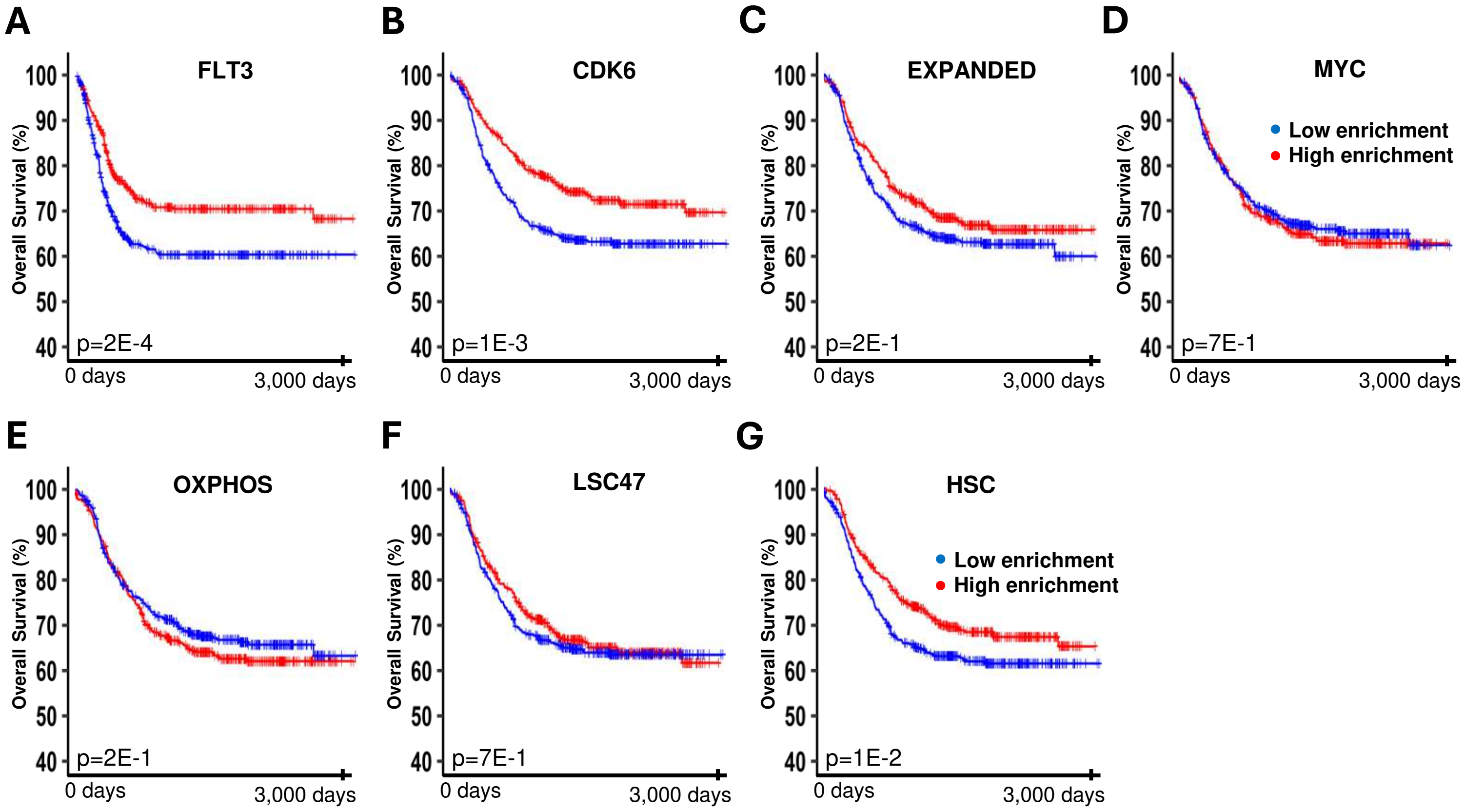

Figure S7

AAML1031 FLT3-ITD Negative KMT2A rearanged (n=234)

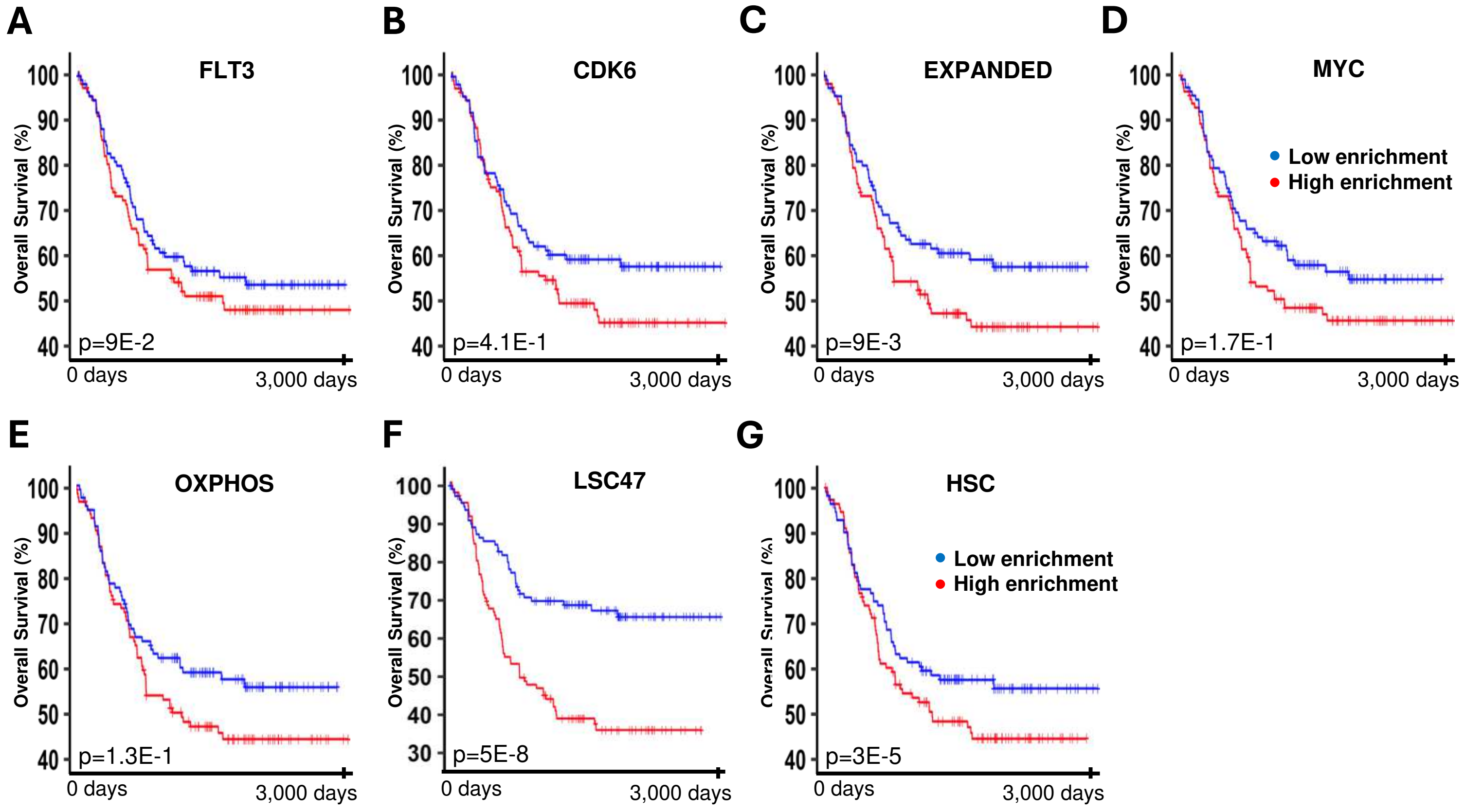

Figure S8

AAML1031 FLT3-ITD Negative Inversion Chr16 (n=96)

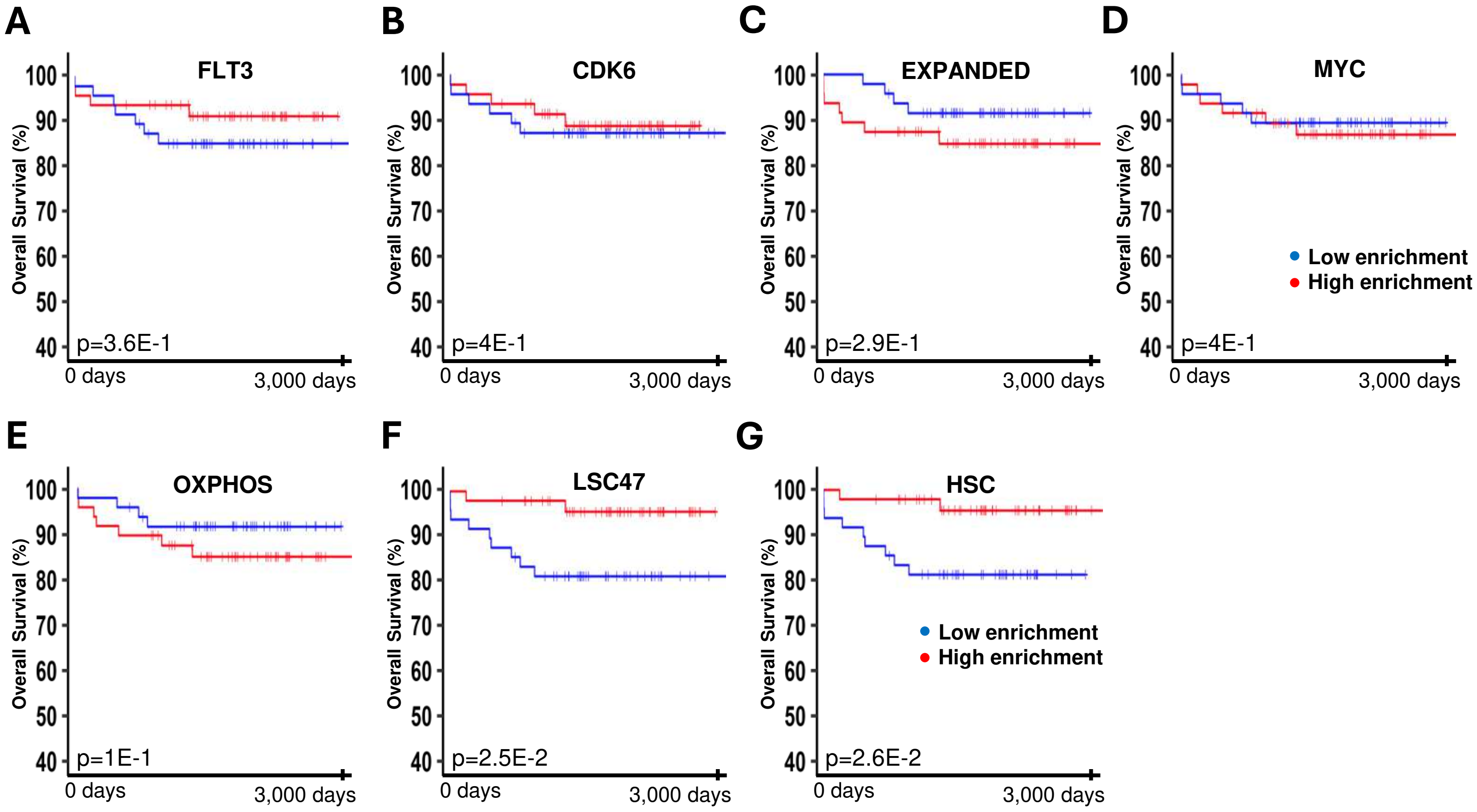

Figure S9

AAML1031 FLT3-ITD Negative *RUNX1* translocation (n=143)

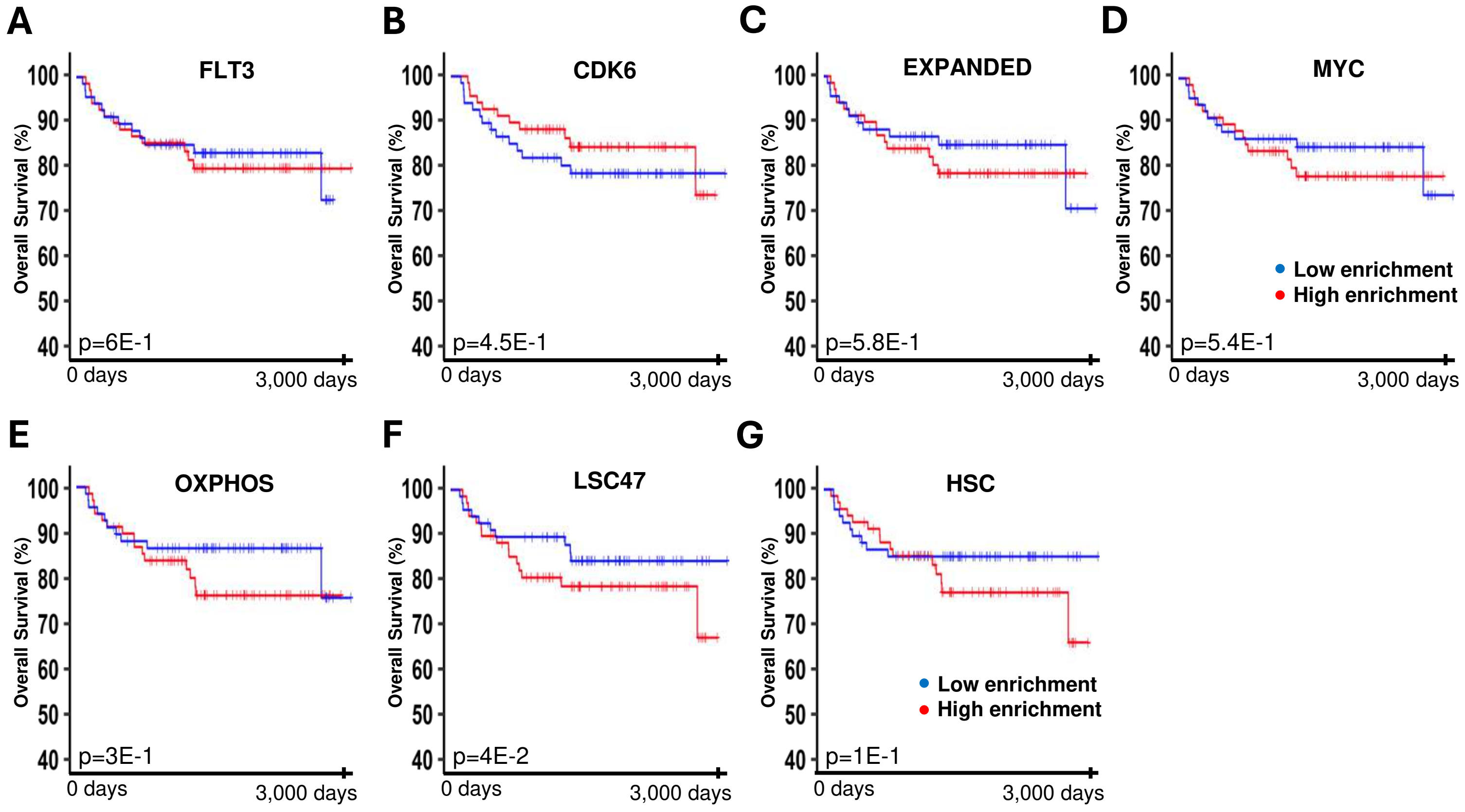

Figure S10

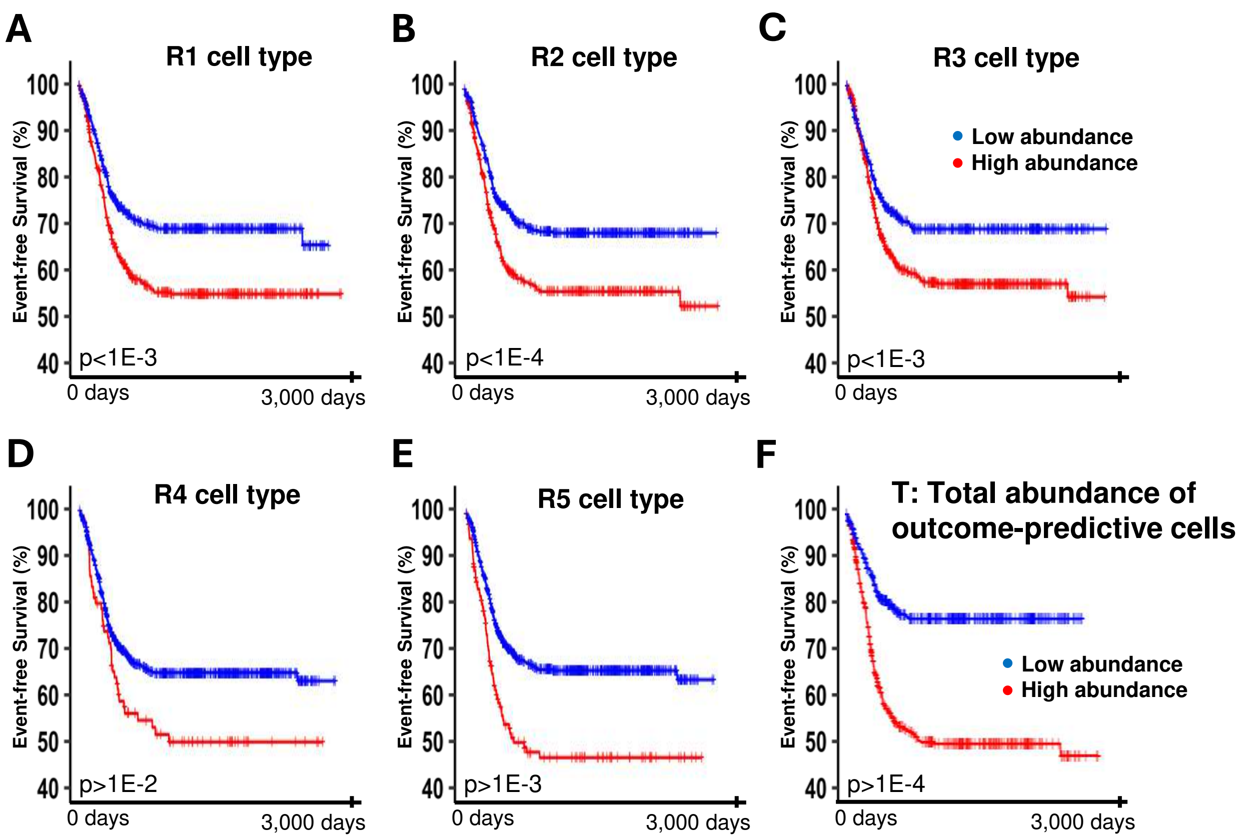
A
R1 cell type
Event-free Survival (%)
100
90
80
70
60
50
40
p<1E-3
0 days
3,000 days
B
R2 cell type
Event-free Survival (%)
p<1E-4
0 days
3,000 days
C
R3 cell type
Event-free Survival (%)
Low abundance
High abundance
p<1E-3
0 days
D
R4 cell type
Event-free Survival (%)
p>1E-2
0 days
3,000 days
E
R5 cell type
Event-free Survival (%)
p>1E-3
0 days
3,000 days
F
T: Total abundance of outcome-predictive cells
Event-free Survival (%)
Low abundance
High abundance
p>1E-4
0 days
3,000 days

Figure S11

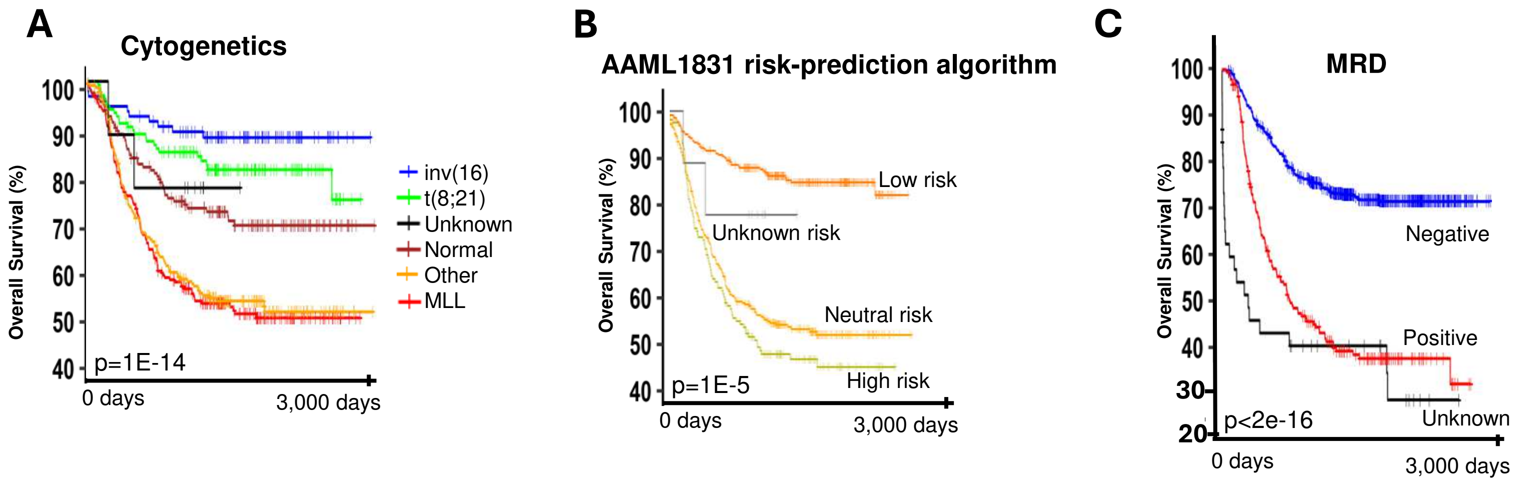
A
Cytogenetics
Overall Survival (%)
100
90
80
70
60
50
40
p=1E-14
0 days
3,000 days
inv(16)
t(8;21)
Unknown
Normal
Other
MLL
B
AAML1831 risk-prediction algorithm
Overall Survival (%)
100
90
80
70
60
50
40
Low risk
Unknown risk
Neutral risk
High risk
p=1E-5
0 days
3,000 days
C
MRD
Overall Survival (%)
100
90
80
70
60
50
40
30
20
Negative
Positive
Unknown
p<2e-16
0 days
3,000 days

Figure S12

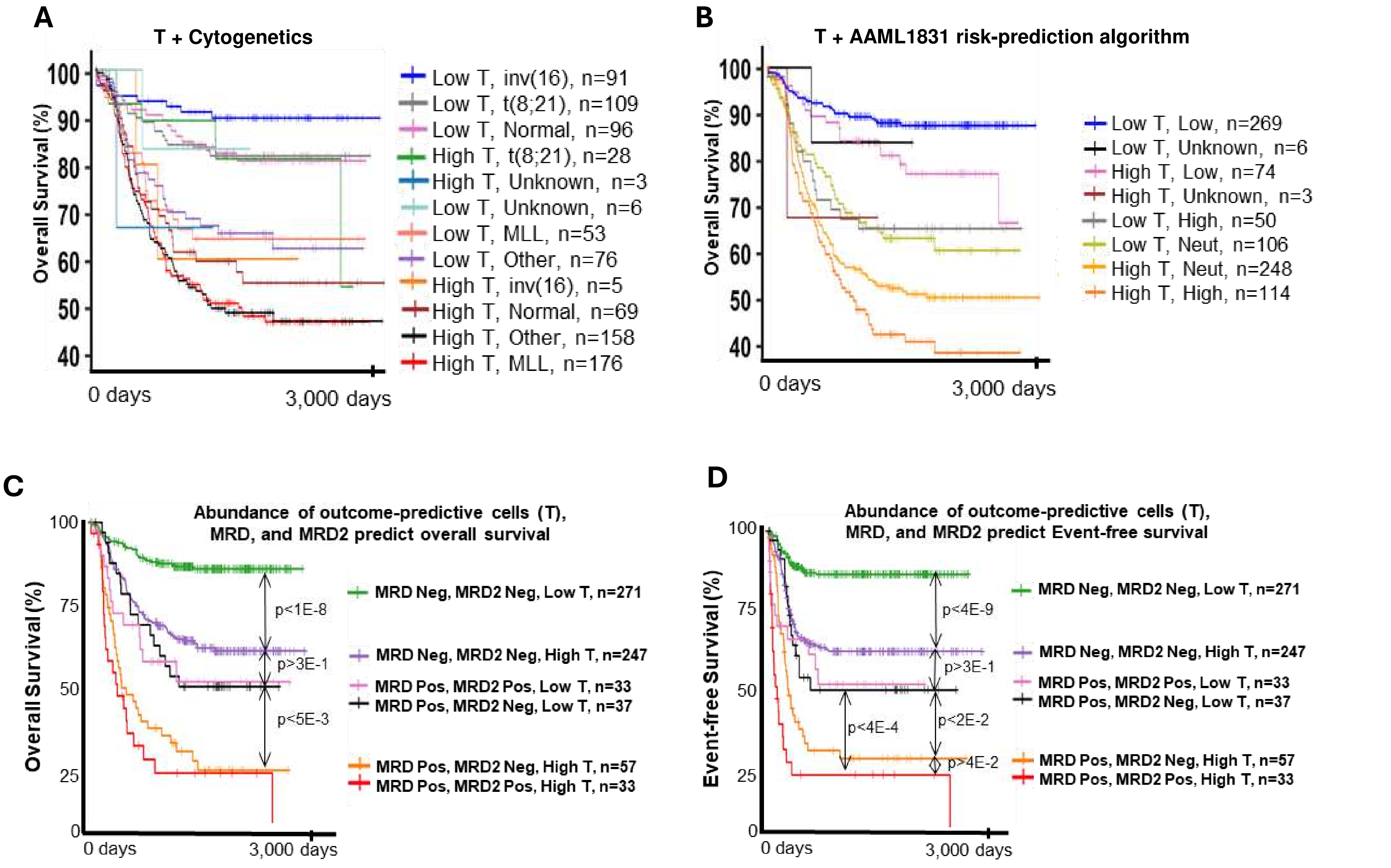
A
T + Cytogenetics
Overall Survival (%)
100
90
80
70
60
50
40
0 days
3,000 days
Low T, inv(16), n=91
Low T, t(8;21), n=109
Low T, Normal, n=96
High T, t(8;21), n=28
High T, Unknown, n=3
Low T, Unknown, n=6
Low T, MLL, n=53
Low T, Other, n=76
High T, inv(16), n=5
High T, Normal, n=69
High T, Other, n=158
High T, MLL, n=176
B
T + AAML1831 risk-prediction algorithm
Overall Survival (%)
100
90
80
70
60
50
40
0 days
3,000 days
Low T, Low, n=269
Low T, Unknown, n=6
High T, Low, n=74
High T, Unknown, n=3
Low T, High, n=50
Low T, Neut, n=106
High T, Neut, n=248
High T, High, n=114
C
Abundance of outcome-predictive cells (T),
MRD, and MRD2 predict overall survival
Overall Survival (%)
100
75
50
25
0
0 days
3,000 days
p<1E-8
p>3E-1
p<5E-3
MRD Neg, MRD2 Neg, Low T, n=271
MRD Neg, MRD2 Neg, High T, n=247
MRD Pos, MRD2 Pos, Low T, n=33
MRD Pos, MRD2 Neg, Low T, n=37
MRD Pos, MRD2 Neg, High T, n=57
MRD Pos, MRD2 Pos, High T, n=33
D
Abundance of outcome-predictive cells (T),
MRD, and MRD2 predict Event-free survival
Event-free Survival (%)
100
75
50
25
0
0 days
3,000 days
p<4E-9
p>3E-1
p<4E-4
p<2E-2
p>4E-2
MRD Neg, MRD2 Neg, Low T, n=271
MRD Neg, MRD2 Neg, High T, n=247
MRD Pos, MRD2 Pos, Low T, n=33
MRD Pos, MRD2 Neg, Low T, n=37
MRD Pos, MRD2 Neg, High T, n=57
MRD Pos, MRD2 Pos, High T, n=33

Figure S13

A

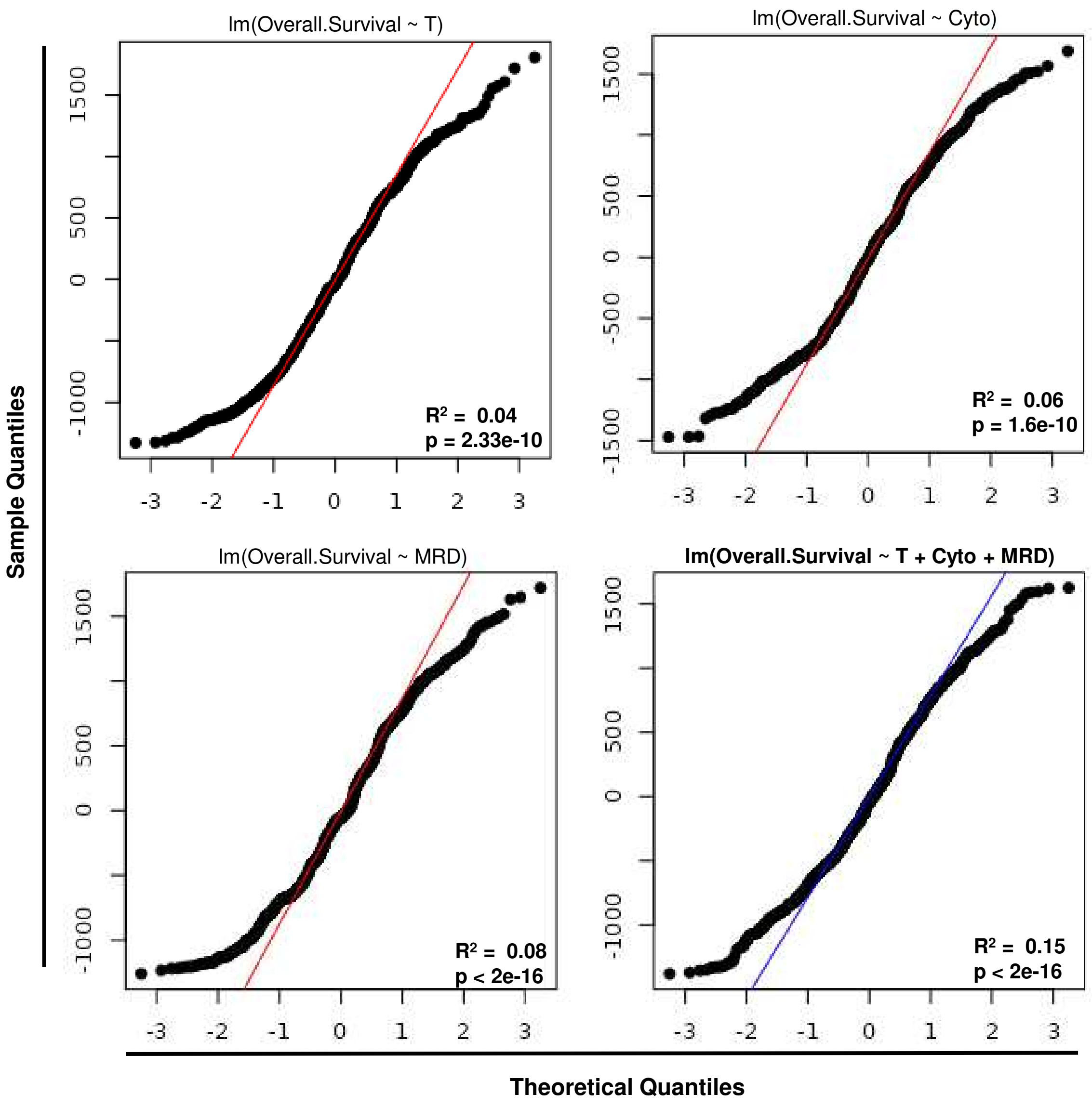

B

	coef	exp(coef)	se(coef)	z	p
T (median, 7%)	0.2381	1.2688	0.0577	4.126	3.69E-05
Inv 16	-0.6834	0.5049	0.3396	-2.013	0.04415
MLL	0.6607	1.9362	0.1814	3.643	0.000269
Other cytogenetics	0.3351	1.3981	0.1821	1.841	0.065693
Unknown cytogenetics	-0.3159	0.7291	0.7278	-0.434	0.664191
t(8;21)	-0.3927	0.6753	0.25	-1.57	0.116324
MRD-Unknown	1.4407	4.2238	0.223	6.46	1.04E-10
MRD-Pos	1.0234	2.7826	0.1282	7.984	1.42E-15

Figure S14

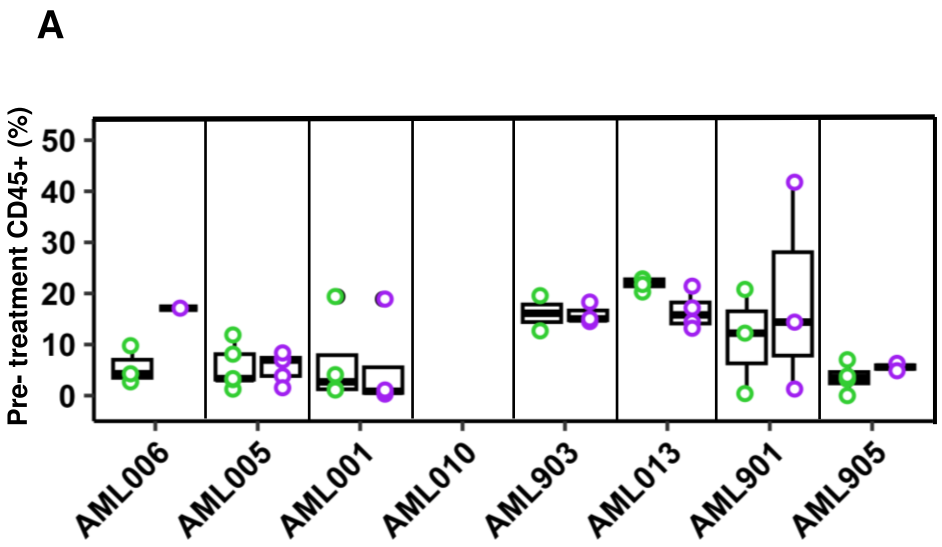

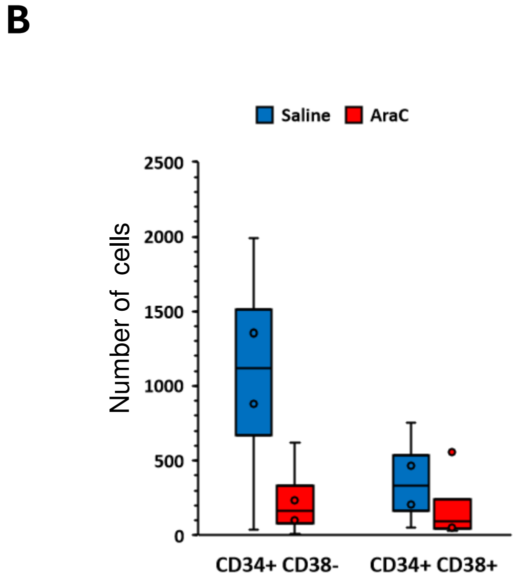

Total number of $CD34^{++}CD38^{-}$ - and $CD34^{+}$+$CD38^{+}$+ human AML cells in the population of residual human $CD45^{+}$+$CD33^{+}$+ cells from AraC-treated PDXs compared to vehicle (saline)-treated xenografted mice.

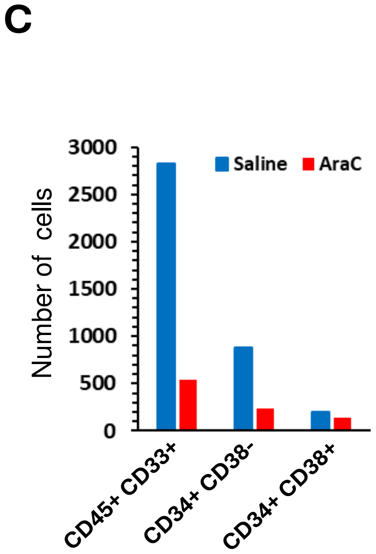

Figure S15
